# Gut microbiome-driven longevity mechanisms in giant pandas: age-associated gut microbial shifts, functional pathways, and probiotic insights

**DOI:** 10.1093/cz/zoaf074

**Published:** 2025-11-21

**Authors:** Feiyun Huang, Xiwei Jin, Jiawang Xu, Honglin Wu, Maoyuan Gan, Wenjingyi Chang, Qihao Tan, Yuhang Wang, Rengui Li, Desheng Li, Xiuyue Zhang

**Affiliations:** Key Laboratory of Bio-Resources and Eco-Environment (Ministry of Education), College of Life Sciences, Sichuan University, No. 24 South Section 1, Yihuan Road, Chengdu 610065, Sichuan, China; China Conservation and Research Center for the Giant Panda, Key Laboratory of SFGA on The Giant Panda, No. 1375 Xiongmao Avenue, Chenghua District, Chengdu 610057, Sichuan, China; School of Life Sciences, Nanchang University, No. 999 Xuefu Avenue, Honggutan New District, Nanchang 330031, Jiangxi Province, China; China Conservation and Research Center for the Giant Panda, Key Laboratory of SFGA on The Giant Panda, No. 1375 Xiongmao Avenue, Chenghua District, Chengdu 610057, Sichuan, China; Key Laboratory of Bio-Resources and Eco-Environment (Ministry of Education), College of Life Sciences, Sichuan University, No. 24 South Section 1, Yihuan Road, Chengdu 610065, Sichuan, China; Key Laboratory of Bio-Resources and Eco-Environment (Ministry of Education), College of Life Sciences, Sichuan University, No. 24 South Section 1, Yihuan Road, Chengdu 610065, Sichuan, China; Key Laboratory of Bio-Resources and Eco-Environment (Ministry of Education), College of Life Sciences, Sichuan University, No. 24 South Section 1, Yihuan Road, Chengdu 610065, Sichuan, China; Key Laboratory of Bio-Resources and Eco-Environment (Ministry of Education), College of Life Sciences, Sichuan University, No. 24 South Section 1, Yihuan Road, Chengdu 610065, Sichuan, China; China Conservation and Research Center for the Giant Panda, Key Laboratory of SFGA on The Giant Panda, No. 1375 Xiongmao Avenue, Chenghua District, Chengdu 610057, Sichuan, China; China Conservation and Research Center for the Giant Panda, Key Laboratory of SFGA on The Giant Panda, No. 1375 Xiongmao Avenue, Chenghua District, Chengdu 610057, Sichuan, China; Key Laboratory of Bio-Resources and Eco-Environment (Ministry of Education), College of Life Sciences, Sichuan University, No. 24 South Section 1, Yihuan Road, Chengdu 610065, Sichuan, China; The Conservation of Endangered Wildlife Key Laboratory of Sichuan Province, College of Life Sciences, Sichuan University, No. 24 South Section 1, Yihuan Road, Chengdu 610065, Sichuan, China

**Keywords:** ailuropoda melanoleuca, enterotype, gut microbiome, longevity, probiotics

## Abstract

Microbiome-mediated longevity mechanisms in conservation wildlife remain largely uncharacterized, particularly in species with specialized diets where host–microbe coevolution plays essential compensatory roles. We conducted cross-sectional metagenomic profiling of gut microbiota in 82 captive giant pandas (*Ailuropoda melanoleuca*) spanning juvenile, adult, and geriatric cohorts. The cohort included 14 long-lived individuals (≥25 years). Our approach integrated enterotype analysis, functional pathway characterization, and lactic acid bacteria (LAB) isolation. Age-specific compositional shifts occurred throughout the lifespan, characterized by reciprocal dominance between Bacillota (declining from 84.77% to 10.26%) and Pseudomonadota (increasing from 12.74% to 81.86%). *Streptococcus* dominated adolescent microbiomes, followed by a transition to cellulose-degrading taxa in young adults. Enterotype analysis revealed 4 distinct community types with age-dependent succession patterns. Long-lived pandas exhibited elevated abundance of *Akkermansia muciniphila*, *Bifidobacterium longum*, *Lactobacillus intestinalis*, and *Ruminococcus gnavus*. Functional profiling revealed age-dependent shifts from nucleotide and amino acid biosynthesis in young animals to lipid metabolism and amino acid degradation in aged individuals. Carbohydrate-active enzyme analysis demonstrated enrichment of cellulolytic enzymes (such as GH3, GH5, and GH9) in long-lived adults. Biosynthetic gene cluster profiling revealed progressive age-associated accumulation of genes encoding NRPS, RiPP-like, arylpolyene, and terpene biosynthetic pathways. Culture-based isolation from fecal samples of long-lived giant pandas yielded 98 LAB strains representing 8 species (*Lactobacillus johnsonii*, *Limosilactobacillus reuteri*, *L. intestinalis*, among others), including 89 non-hemolytic strains that were sensitive to 6 common antibiotics. These findings suggest potential cross-species conservation of microbiome-longevity mechanisms, with implications for understanding physiological adaptations in specialist herbivores and applications in conservation medicine.

## Graphical Abstract

**Graphical Abstract:**
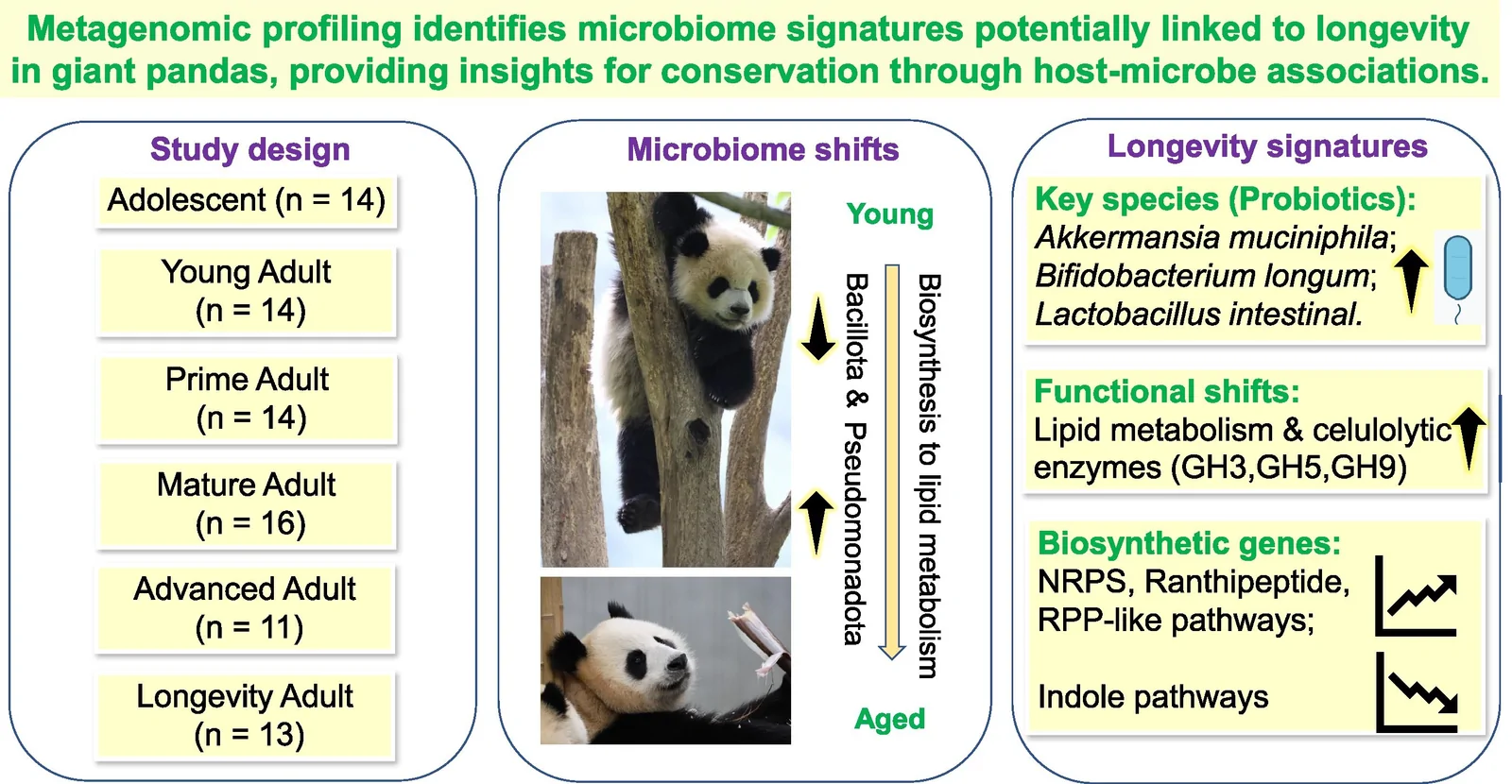

The gut microbiome functions as a “forgotten endocrine organ” in humans and animals. It plays pivotal roles in host health maintenance, nutritional metabolism, and immune regulation (Sonnenburg and Fischbach 2011). Disruption of host-associated microbial communities through habitat degradation, captive management, or medical interventions has been shown to compromise threatened species' health and survival (West et al. 2019). Microbiome integrity has emerged as a critical consideration in conservation practice, with over 13,000 animal species currently classified as threatened globally (Stumpf et al. 2016). Captive breeding programs often expose animals to sterile environments, simplified diets, and antibiotic treatments. These alterations to host microbiomes are associated with poor health outcomes and reproductive failures (Wasimuddin et al. 2017). Host-associated microbial communities profoundly influence immunity and pathogen resistance, with their plasticity enabling microbiome-targeted conservation interventions (Alexander et al. 2024). Probiotic bioaugmentation has proven effective in threatened species. *Janthinobacterium lividum* treatment increased the of *Batrachochytrium dendrobatidis*-challenged boreal toads (*Anaxyrus boreas*) by 40% (Kueneman et al. 2016). Similarly, *Pseudomonas fluorescens* treatment increased overwinter survival of bats with white-nose syndrome more than 5-fold (Hoyt et al. 2019). These results demonstrate microbiome manipulation as a viable conservation strategy for mitigating pathogen-driven declines.

Beyond conservation applications, advances in omics technologies have established that gut microbiota are involved in regulating aging trajectories, positioning microbial communities centrally in healthy aging research (Wilmanski et al. 2021). Shared microbiota characteristics between longevity populations and younger individuals—manifested through elevated community evenness and predominant *Bacteroides* enterotype—have been documented (Pang et al. 2023), underscoring the significance of maintaining microbial ecological balance for healthspan extension. Aging microbiome research has been largely confined to humans and traditional model organisms, overlooking non-model animals as products of adaptive evolution that provide unique perspectives for understanding microbiome–host longevity interactions. Dietary strategies, energy metabolism patterns, and lifespan phenotypes developed through phylogenetic processes render animal gut microbiomes rich sources of molecular ecological information, constituting natural experimental systems for microbe-host coevolution studies (Henry et al. 2021). Phylogenetic compositional similarities between naked mole-rat (*Heterocephalus glaber*, Rüppell, 1842) gut microbiomes and human centenarians, including enriched low-abundance Mogibacteriaceae, support the use of naked mole-rats as mammalian models in aging research (Debebe et al. 2017). Caste differentiation in the honey bee (*Apis mellifera*, Linnaeus, 1758) demonstrates parallel evidence. Genetically identical yet phenotypically divergent workers and queens exhibit structural and functional microbiome differentiation, with queen microecosystems maintaining elevated *Lactobacillus* and *Bifidobacterium* populations alongside downregulated oxidative stress responses (Wang et al. 2022). Cross-phylogenetic comparative analyses suggest evolutionarily conserved mechanisms linking microbial communities and host lifespan phenotypes; however, wildlife microbiome functional studies remain exploratory compared with human research, particularly lacking systems biology integration of longevity phenotypes and dietary adaptations.

Giant pandas (*Ailuropoda melanoleuca*, David, 1869) exemplify an evolutionary paradox: carnivorans specialized for bamboo consumption yet lacking endogenous cellulases. Their gut microbiota partially compensate for this deficit, showing preferential degradation of hemicellulose over cellulose (Zhu et al. 2011; Zhang et al. 2018). However, this microbial contribution remains limited—cellulases and endohemicellulases constitute only 2% of microbial glycoside hydrolases (Zhu et al. 2011). This incomplete metabolic adaptation makes giant pandas a valuable model for studying host–microbe interactions under extreme dietary constraints.

Captivity profoundly alters the gut microbial ecosystem. Captive populations exhibit significantly lower gut microbiota diversity and altered composition compared with wild populations (Guo et al. 2019). Gastrointestinal pathologies represent a primary mortality cause in both wild and captive pandas (Janssen et al. 2006). Seasonal dietary shifts driven by bamboo phenology are associated with chronic gastrointestinal disturbances characterized by abdominal pain, inappetence, and mucoid stool (Williams et al. 2016). These findings suggest that microbiome composition may influence health outcomes and potentially longevity in giant pandas, though long-term evidence remains limited.

Limited sample sizes and predominantly descriptive approaches have constrained research on the giant panda microbiome. Studies over the past decade have characterized microbial composition and predicted functional potential (Zhu et al. 2011; Zhang et al. 2018; Guo et al. 2019), yet experimental demonstrations of how specific microbes or metabolites impact host physiology remain scarce. Although debates continue regarding microbial contributions to bamboo fiber digestion, the broader question of whether and how the microbiome influences host healthspan remains largely unexplored. Systematic investigation across 3 dimensions is essential: establishing enterotype classification systems to characterize microbiome compositional states; identifying candidate longevity-associated microbes and assessing functional pathway conservation across species; and evaluating whether endangered species harbor distinct beneficial microorganisms that may inform conservation strategies. These objectives require integrating expanded sampling with functional genomics and culture-based validation to deepen understanding of host-microbe interactions under anthropogenic pressure.

## Materials and methods

### Sample collection

Fecal samples (*n* = 82) were collected from captive giant pandas housed at the China Conservation and Research Center for the Giant Panda and its affiliated facilities (Dujiangyan, Ya’an Bifengxia, and Wolong Shenshuping) between December 2024 and January 2025. Fresh fecal samples were collected within 5 min of observed defecation from undisturbed animals maintained under continuous observation by personnel positioned at a minimum distance of 5 m. Central portions of fecal matter (5–10 g) were aseptically sampled using sterile, individually wrapped spatulas. Prior to each sampling event, spatulas were surface-disinfected with 70% (v/v) ethanol and allowed to air-dry completely (minimum 30 s) to prevent residual alcohol from interfering with downstream DNA extraction. A double-gloving protocol was employed throughout collection, with inner latex gloves covered by outer polyethylene gloves. Outer glove surfaces were disinfected with 70% ethanol between individual animal observations. Outer gloves were removed immediately following each collection and replaced between consecutive samples to prevent cross-contamination. Individual samples were transferred to sterile 50-mL polypropylene tubes (certified DNA-free; Corning) without pooling. Metadata were recorded for each sample, including individual identification code, sex, age, birth origin (captive-born or wild-born), health status, and precise collection time (Supplementary Table S1). Samples were transported on ice within 1 h of collection and subsequently stored at −80 °C until analysis.

The study cohort comprised 33 males and 49 females ranging from 2 to 33 years of age, including 75 captive-born and 7 wild-born individuals. Age stratification was established based on documented physiological and reproductive milestones in giant panda life history, including sexual maturity onset (4–6 years), peak reproductive performance (6–15 years), and late reproductive period with gradual decline (16–20 years). Progressive senescence beyond 20 years is characterized by altered metabolic profiles, immune system dysfunction, and increased susceptibility to age-related diseases (Zhu et al. 2001; Swaisgood et al. 2020; Zhang et al. 2022; Luo et al. 2025).

Based on these biological parameters, we implemented 6 age categories. Adolescent (1.5–5 years, *n* = 14) encompassed the pre-reproductive and pubertal transition period. Young adult (6–10 years, *n* = 14) and prime adult (11–15 years, *n* = 14) represented early and peak reproductive maturity, respectively. Mature adult (16–20 years, *n* = 16) was characterized by late reproductive capacity with gradual decline, whereas advanced adult (21–24 years, *n* = 10) marked reproductive senescence and age-related physiological deterioration. Longevity adult (≥ 25 years, *n* = 14) comprised individuals approaching or exceeding the 75th percentile of captive giant panda lifespan, given that captive giant pandas typically live 25–30 years, with maximum recorded longevity reaching 38 years (Jin et al. 2012; You et al. 2019). These age divisions align with documented physiological transitions, as blood-based immune and metabolic markers change significantly across age categories (Huang et al. 2020; Liu et al. 2023a).

To support age-appropriate physiological needs across these life stages, animals received standardized diets comprising concentrate feed (formulated pellets of corn, rice, soybeans, egg powder, and mineral-vitamin premix), bamboo, and fruits and vegetables. Daily rations for adolescents (1.5–5 years) consisted of 1–2 kg concentrate feed, 3–8 kg bamboo (10–30 kg offered), and 1–3 kg fruits and vegetables. Post-adolescent individuals (≥6 years) received 2 kg concentrate feed, 5–8 kg bamboo (30–40 kg offered), and 2–5 kg fruits and vegetables. Bamboo and water were available ad libitum.

### Fecal metagenomic sequencing

Total DNA was extracted from fecal samples using the QIAamp PowerFecal Pro DNA Kit (Qiagen, Hilden, Germany) following the manufacturer's protocol. Samples were homogenized using a Wonbio-96 high-throughput tissue grinder (Shanghai Wonbio Biotechnology Co., Ltd., Shanghai, China) at 60 Hz for 5 min. DNA concentration and purity were determined using a NanoDrop 2000 spectrophotometer (Thermo Fisher Scientific, Waltham, MA, USA), and DNA integrity was verified by 1% agarose gel electrophoresis.

DNA was fragmented to approximately 350 bp using a Covaris S220 Focused-ultrasonicator (Covaris, Woburn, MA, USA), followed by size selection and purification with Agencourt AMPure XP magnetic beads (Beckman Coulter, Brea, CA, USA). Metagenomic libraries were constructed using the TruSeq Nano DNA LT Sample Preparation Kit (Illumina, San Diego, CA, USA) according to the manufacturer's instructions. Paired-end sequencing (2 × 150 bp) was performed on an Illumina NovaSeq 6000 platform, generating an average of 48 million read pairs (7 Gb of raw data) per sample. All library preparation and sequencing procedures were conducted by Shanghai OE Biotech Co., Ltd. (Shanghai, China).

### Metagenomic analysis

Raw sequencing data underwent quality control using Fastp (v0.20.1) to remove adapter sequences and low-quality reads (Chen 2023). Host-derived contamination was eliminated by mapping remaining reads to the giant panda reference genome (ASM200744v3) using Bowtie2 (v2.4.4) (Langmead and Salzberg 2012). Species annotation was performed with Kraken2 (v2.1.3) against the Kraken2 PlusPF database (69GB) containing bacterial, archaeal, and viral reference sequences. Read counts at multiple taxonomic ranks were estimated using Bracken (v2.9), which probabilistically redistributed Kraken2-classified reads through Bayesian re-estimation (Lu et al. 2017; Wood et al. 2019). Estimated read counts were converted to relative abundances to normalize for sequencing depth.

Functional profiling of filtered metagenomic sequences was conducted using HUMAnN3 (v3.7) with ChocoPhlAn and UniRef90 databases (index version mpa_vOct22_CHOCOPhlAnSGB_202212) (Suzek et al. 2007; Beghini et al. 2021). Gene functions were predicted and metabolic pathway abundances were quantified through sequence alignment to known microbial gene families. Co-assembly was performed using MEGAHIT (v1.2.9) with default parameters (–k-list 21, 29, 39, 59, 79, 99, 119, 141; –min-count 2; -m 0.9) (Li et al. 2015). MEGAHIT's multi-k strategy progressively refines the assembly from small to large k-mer sizes, improving sensitivity to low-abundance genomes at smaller *k* values whereas enhancing contiguity and specificity at larger *k* values. After assembly, contigs shorter than 500 bp were excluded using seqkit (v2.0.0) to reduce fragmentation noise prior to downstream analyses (Shen et al. 2024).

Open reading frames (ORFs) were predicted using Prodigal (v2.6.3) in metagenomic mode (Hyatt et al. 2010). Non-redundant gene catalogs were constructed using CD-HIT-EST clustering at 95% sequence identity and 90% coverage thresholds (Fu et al. 2012). Gene abundance was quantified using Salmon (v1.10.3), which maps sample reads to non-redundant gene sequences to generate transcripts per million (TPM) values and read counts (Patro et al. 2017). Carbohydrate-active enzyme (CAZyme) annotation was performed using eggNOG-mapper (Cantalapiedra et al. 2021) with the DIAMOND algorithm (Buchfink et al. 2015). CAZy family abundance matrices were generated by integrating Salmon-derived abundance data with CAZy functional annotations using summarizeAbundance.py. These matrices were then mapped to the CAZy classification system to analyze microbial carbohydrate metabolism potential (Liu et al. 2023b).

Secondary metabolite biosynthetic gene clusters (BGCs) were identified from assembled contigs using antiSMASH (v8.0) with bacterial taxon specification and Prodigal gene-finding; all other parameters were set to default values (Blin et al. 2023). BGC classes and counts were extracted from antiSMASH output files for each sample. To quantify BGC abundance at the community level, contig-level TPM values were obtained by mapping quality-filtered reads to sample-specific contig indices using Salmon as described above. The abundance of each BGC class per sample was calculated as the sum of products between contig abundance (TPM) and the number of BGCs of that class on each contig. This approach captures both the presence and expression level of BGC-containing contigs.

### Microbial diversity and differential analysis across age groups

Linear discriminant analysis effect size (LEfSe) analysis was performed on microbial abundance data using the microeco package in R (v4.4.3) (Liu et al. 2021). LEfSe assigns each biomarker to the group where it exhibits the highest LDA score, representing its primary age-group association (Segata et al. 2011). Dual screening criteria were applied (LDA > 2 at genus and species level, *P* < 0.05) to identify age-specific enriched biomarker taxa. False discovery rate (FDR) correction controlled for multiple comparisons. Statistical significance threshold was set at *P* < 0.05. All analytical results were visualized using ggplot2 package (v3.3.5).

Dirichlet multinomial mixture (DMM) clustering was executed on genus-level microbial abundance data using the DirichletMultinomial package (v1.36.0) in R (Holmes et al. 2012). Model fit optimization was assessed through Laplace estimation across different cluster numbers (*k* = 1 to 10) to determine the optimal number of enterotypes. Sample distribution patterns across enterotypes were analyzed for each age stage, with ecological interpretation based on dominant genera characteristics revealing intrinsic associations between enterotypes and host physiological age.

Principal coordinate analysis (PCoA) dimensionality reduction was applied to MetaCyc (HUMAnN3-derived) and CAZyme (assembly-annotation-derived) pathway abundance data using the vegan package (v2.5-7). This analysis constructed Bray-Curtis distance-based functional composition similarity matrices (Dixon 2003). Permutational multivariate analysis of variance (PERMANOVA, permutations = 999) assessed statistical significance of functional composition differences between age groups and explained variance proportions (*R*^2^). Significantly different metabolic pathways underwent LDA analysis (LDA > 2 and *P* < 0.05) to identify age-specific enriched functional pathways, subsequently categorized by biological significance.

To address potential confounding by host variables, we performed PERMANOVA using Type III marginal tests (adonis2 function in vegan package) to partition variance in both genus-level taxonomic composition and pathway abundance data among life stage, sex, sampling location, and birth origin (Dixon 2003). This approach enabled us to evaluate the independent contribution of life stage while accounting for other host factors. Both univariate models testing each variable individually and a full multivariate additive model incorporating all variables simultaneously were constructed to assess the robustness of life-stage-associated patterns after controlling for covariates. Variance explained was quantified by the coefficient of determination (*R*^2^).

To identify individual taxa and pathways driving life-stage-associated shifts, we performed multivariable regression modeling using MaAsLin3 (Nickols et al. 2024). MaAsLin3 implements generalized linear models with fixed effects for life stage, sex, location, and birth origin, enabling feature-wise association testing adjusted for host variables. Genus-level taxonomic composition and MetaCyc pathway abundances were normalized using total sum scaling (TSS) and subsequently log-transformed prior to modeling. Differentially abundant features were identified based on both coefficient magnitude and false discovery rate (*P* < 0.05, Benjamini–Hochberg correction).

Key intestinal species associated with host health and longevity (*Ruminococcus gnavus*, *A. muciniphila*, *B. longum*, *Lactobacillus intestinalis*) were selected based on literature reports (Xia et al. 2020; Wu et al. 2022; Wang et al. 2023; Zeng et al. 2023; Ku et al. 2024; Andreu-Sánchez et al. 2025; Sun et al. 2025). Centered log-ratio (CLR) transformation corrected compositional bias in abundance data. Age-dependent abundance patterns were visualized through boxplots, with Kruskal–Wallis test evaluating significance (*P* < 0.05) to explore ecological implications of potential longevity-associated bacteria.

### Isolation of lactic acid bacteria derived from giant pandas

Lactic acid bacteria (LAB) were isolated from fresh fecal samples (1 g) of giant pandas (aged ≥ 25 years) through serial dilution (10⁻¹ to 10⁻⁶) in sterile saline (9 mL of 0.9% NaCl). Dilutions were plated on MRS agar supplemented with 1% (w/v) CaCO₃ and incubated anaerobically at 37 °C for 24 h. Calcium carbonate incorporation facilitates rapid visual screening of acid-producing strains through formation of clearance zones surrounding colonies. Colonies with clearance zones exceeding 2 mm in diameter were selected for subsequent characterization. This approach leverages the fermentative capacity of LAB to produce organic acids—predominantly lactic acid—which lower intestinal pH and generate bioactive metabolites. These metabolic activities confer multifaceted probiotic properties, including antimicrobial activity, intestinal barrier enhancement, and immune modulation, all critical for giant panda gut homeostasis (Huang et al. 2024). Although this calcium carbonate-based method may not efficiently recover acetate-producing *Bifidobacterium*, it enables targeted isolation of LAB strains with documented health-promoting properties relevant to animal conservation.

Following incubation, colony morphology and characteristics were examined. Individual colonies exhibiting distinct morphological features, varied pigmentation, and prominent calcium dissolution zones were selected for purification through 2 consecutive streak plating procedures on MRS agar. Purified isolates were transferred using sterile inoculation loops into cryogenic vials containing 600 μL MRS broth medium, cultured overnight at 37 °C with agitation, mixed with 400 μL 50% glycerol, and preserved at −80 °C.

Identification of bacterial isolates was achieved via 16S rRNA gene sequencing. Amplification employed universal primers 27F (5′-AGAGTTTGATCCTGGCTCAG-3′) and 1492R (5′-GGTTACCTTGTTACGACTT-3′). Reaction components (25 μL total) consisted of template DNA (1 μL), forward and reverse primers (1 μL each), Taq MasterMix (12.5 μL), and double-distilled water (9.5 μL). Thermal cycling parameters were established as follows: initial denaturation (95 °C, 5 min), 30 amplification cycles (denaturation: 95 °C, 30 s; annealing: 54 °C, 30 s; extension: 72 °C, 45 s), and final extension (72 °C, 10 min) (Huang et al. 2025).

Amplicons were subjected to bidirectional sequencing. Taxonomic assignment was accomplished via BLAST algorithm comparison. Phylogenetic reconstruction employed MEGA 11.0 software, utilizing Clustal-based sequence alignment and neighbor-joining methodology for taxonomic placement determination.

### Antibiotic susceptibility testing

Antibiotic susceptibility profiles of isolated strains against clinically relevant antibiotics were evaluated using the Kirby–Bauer disk diffusion method. Strains stored at −80 °C were reactivated on MRS agar plates, and single colonies were inoculated into MRS broth for 18 h incubation at 37 °C until the logarithmic growth phase. Bacterial cells were harvested by centrifugation (5,000 g, 10 min), washed twice with sterile physiological saline, and adjusted to 0.5 McFarland turbidity standard (OD600 ∼ 0.1, equivalent to 1 × 10^8^ CFU/mL). Standardized bacterial suspensions were uniformly spread onto MRS plates using sterile cotton swabs. After 5 min surface absorption, antibiotic disks containing ceftriaxone, tetracycline, clarithromycin, chloramphenicol, ampicillin, and clindamycin were placed at intervals ≥24 mm. Incubation was performed for 18 h at 37 °C. Zone diameter measurements were subsequently obtained and categorized as susceptible (S), intermediate (I), or resistant (R) based on Clinical and Laboratory Standards Institute (CLSI) criteria. *Lactobacillus rhamnosus* GG ATCC 53103 (LGG) was employed as the reference control strain (Humphries et al. 2021; Huang et al. 2025).

### Hemolytic activity testing

Hemolytic activity of isolated strains was evaluated on Columbia blood agar plates. After 24 h of incubation on MRS plates, single colonies were streaked onto Columbia agar plates supplemented with 5% (v/v) sheep blood. After 48 h of incubation at 37 °C, hemolytic phenotypes were observed and classified based on the characteristics of zones surrounding colonies: α-hemolysis (greenish zones indicating partial hemolysis), β-hemolysis (clear zones indicating complete hemolysis), and γ-hemolysis (absence of zones indicating no hemolytic activity). For each strain, 3 independent experiments were performed. *Staphylococcus aureus* CMCCB 26003 and LGG served as positive and negative controls, respectively. Only strains exhibiting a γ-hemolytic (non-hemolytic) phenotype were considered suitable for further functional characterization. Antibiotic susceptibility and hemolytic activity assays were conducted as preliminary safety screening for probiotic applications. Comprehensive functional characterization of longevity-associated mechanisms (including anti-inflammatory activity, short-chain fatty acid production, and oxidative stress mitigation) was reserved for subsequent mechanistic investigations.

### Statistical analysis for LAB safety characterization

Data from 3 independent replicates were expressed as mean ± standard deviation. Comparisons between test strains and the reference strain (LGG) were performed using Dunnett's test for multiple comparisons against a single control. Statistical significance was set at *P* < 0.05. All analyses were conducted using R (v4.4.3).

## Results

### Data quality control

Metagenomic sequencing of 82 fecal samples from captive giant pandas generated a total of 3.94 billion raw reads (591.2 Gb raw data). After quality filtering, 3.93 billion clean reads (589.1 Gb) were retained, with an average of 47.89 ± 3.48 million clean reads per sample (range: 38.89 to 77.97 million). The quality control process demonstrated exceptionally high data retention, with 99.79% ± 0.39% of raw reads passing quality filters (range: 97.51% to 99.97%). Similarly, 99.53% ± 0.42% of raw bases were retained after filtering (range: 97.51% to 99.83%).

Sequencing quality metrics indicated high-quality data across all samples. The average percentage of bases with Phred quality scores ≥ 20 (Q20) was 98.19% ± 0.32% (range: 97.57% and 99.00%), whereas bases with Q30 averaged 94.95% ± 0.78% (range: 93.48% and 96.88%). These values substantially exceed the typical thresholds for high-quality sequencing data (Q20 > 90%, Q30 > 85%). The GC content across samples averaged 43.63% ± 3.41% (range: 37.83% and 52.07%) (Supplementary Table S2). The relatively narrow interquartile range suggests consistent microbial community composition across samples, whereas the full range indicates natural biological variation among individuals. All samples met or exceeded standard quality criteria for metagenomic analysis, ensuring reliable downstream analyses of the giant panda gut microbiome.

### Microbial community composition analysis

We identified systematic age-dependent microbial succession across 75 phyla, 2,374 genera, and 10,250 species. At the phylum level, Bacillota and Pseudomonadota exhibited reciprocal dominance patterns. Bacillota predominated in the adolescent group (84.77%) and young adult group (76.51%) but declined sharply in prime adults (32.70%) and mature adults (35.40%). Bacillota abundance reached 10.26% in the advanced adult group before partial recovery in longevity adults (17.76%). Conversely, Pseudomonadota increased from 12.74% in adolescents to peak levels of 61.33% and 81.86% during mid-to-late life stages. Uroviricota progressively expanded from 0.38% to 6.12% with aging, whereas Bacteroidota and Actinomycetota showed enrichment in aged individuals (2.84% and 2.08%, respectively) (Figure 1A).

**Figure 1:**
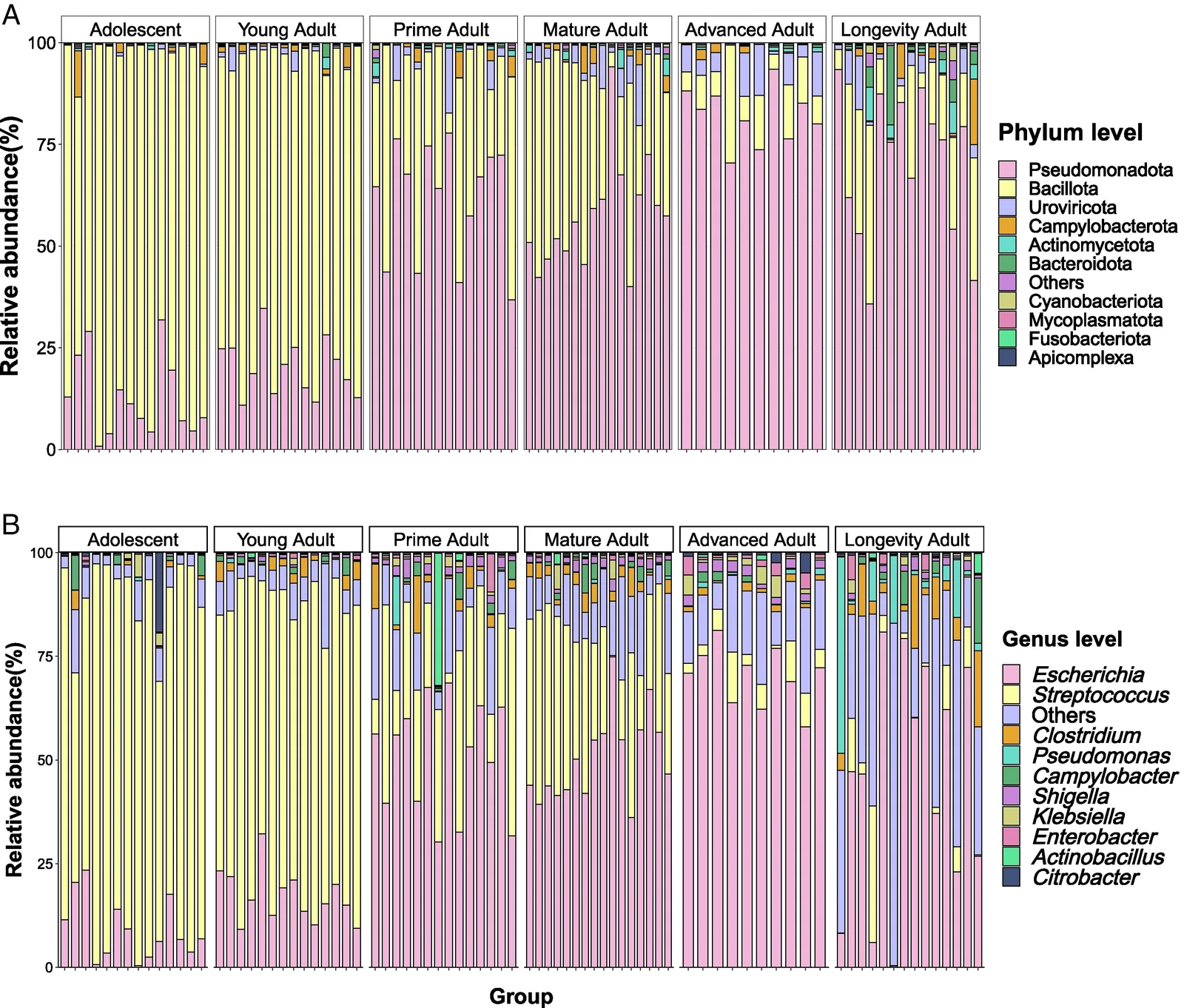
Age-dependent succession of gut microbiota composition in captive giant pandas. (A) Relative abundance of bacterial phyla across 6 age stages showing reciprocal dominance patterns between Bacillota and Pseudomonadota. (B) Genus-level microbial community structure demonstrating the transition from *Streptococcus*-dominated to *Escherichia*-dominated communities with advancing age.

Genus-level dynamics mirrored phylum patterns. *Streptococcus* declined from 80.76% in Adolescents to 5.37% in advanced adults, whereas *Escherichia* rose from 9.05% to 70.25% across the same period. Longevity adults harbored elevated *Clostridium* (5.87%) and *Pseudomonas* (7.09%) (Figure 1B).

At the species level, *Streptococcus alactolyticus* decreased from 68.58% in adolescents to 4.29% in advanced adults, whereas *Escherichia coli* increased from 8.78% to 69.39%. Multiple *Streptococcus* species showed parallel declines (such as *Streptococcus gallolyticus*: 1.57% to 0.14%). Longevity adults exhibited distinctive signatures, including elevated *Aeromonas caviae* (2.59%), *Pseudomonas fluorescens* (2.14%), and enhanced diversity with non-dominant taxa comprising 39.32% of the microbiome (Supplementary Figure S1).

### Age-associated microbial biomarkers

Significant taxonomic differences at the genus level across 6 age cohorts in giant pandas were revealed by LEfSe analysis, identifying 97 differentially abundant genera (LDA > 2, *P* < 0.05). *Streptococcus* emerged as the dominant microbial biomarker in adolescents (LDA = 5.57), accompanied by significant enrichment of LAB, including *Leuconostoc* (3.57), *Enterococcus* (3.33), and *Lactococcus* (3.21). Young adults showed fewer differentially abundant genera, primarily *Ruminococcus* (2.50), *Blautia* (2.04), and *Fremauxvirus* (2.50). *Ruminococcus* species utilize cellulosomes for plant cell wall polysaccharide degradation (Israeli-Ruimy et al. 2017; Weimer 2022). In mature adults, viral genera constituted the characteristic taxonomic signature, notably *Gamaleyavirus* (3.45) and *Skatevirus* (2.89). Advanced adult microbiome was distinctly characterized by *Escherichia* predominance (5.48), with concurrent enrichment of *Shigella* (3.97), *Klebsiella* (3.93), *Citrobacter* (3.86), and *Enterobacter* (3.85). Longevity adults displayed the most diversified microbial signature, with *Pseudomonas* (4.55), *Clostridium* (4.39), and *Aeromonas* (4.22) serving as primary discriminatory markers (Figure 2A).

**Figure 2:**
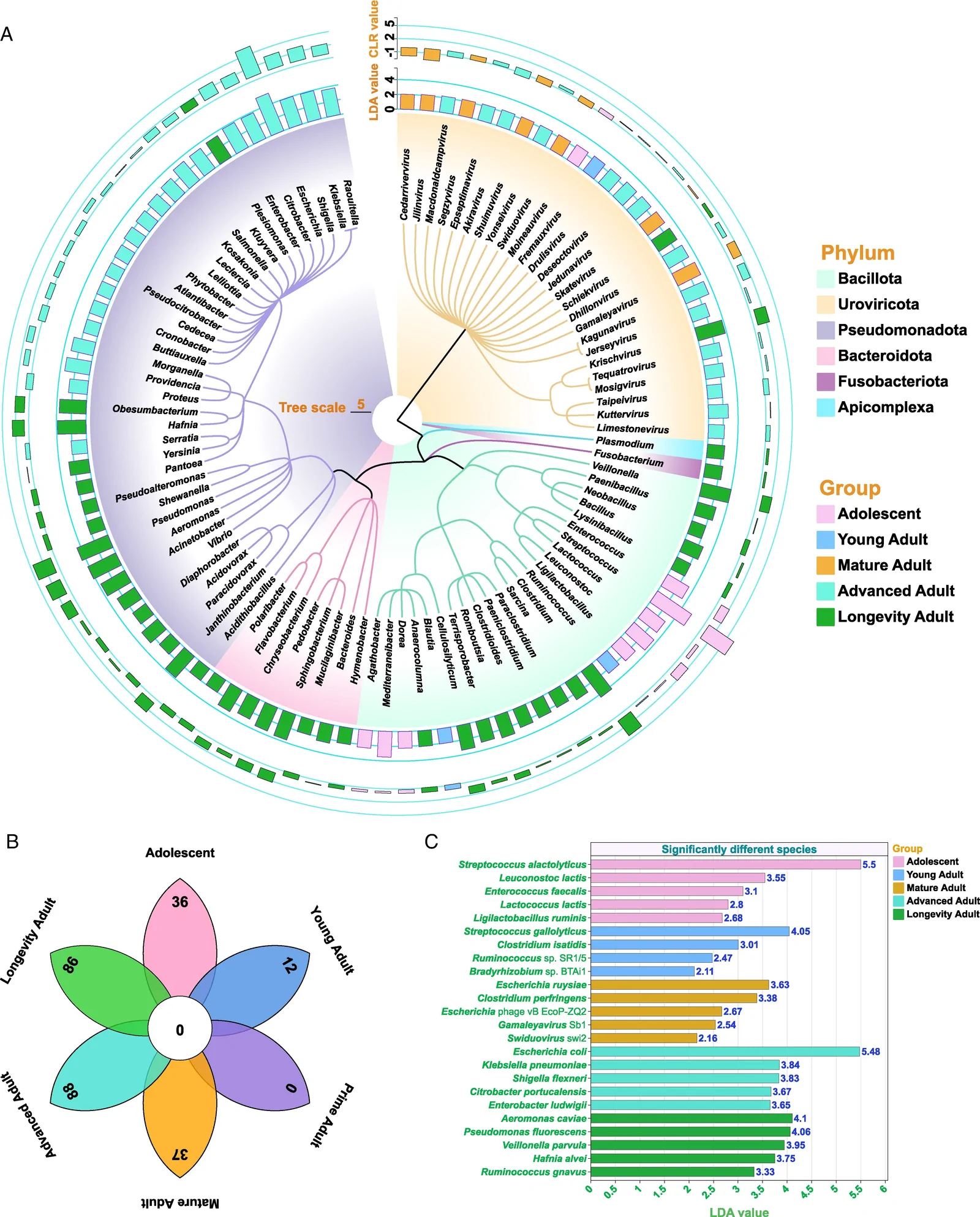
Age-associated microbial biomarkers in captive giant pandas identified through LEfSe analysis. (A) Circular cladogram of genus-level taxa (LDA score > 2) enriched in different age groups. Colored segments denote significantly enriched taxa, with inner bars indicating LDA scores and outer bars displaying CLR-transformed relative abundances. (B) Venn diagram illustrating unique and shared differentially abundant species among age groups. Each species is assigned to the age group where it exhibits the highest discriminatory power (LDA score), representing its strongest association. (C) LDA scores of the most significantly enriched species in each age cohort (LDA > 2, *P* < 0.05).

Species-level LEfSe analysis identified 259 differentially abundant taxa across 6 age cohorts, with marked disparities in cohort-specific enrichment patterns. Maximum taxonomic diversity characterized advanced adults (88 species), followed by longevity adults (86 species) (Figure 2B). *S. alactolyticus* (LDA = 5.50) emerged as the predominant biomarker in adolescents, accompanied by selective enrichment of multiple LAB: *Leuconostoc lactis* (3.55), *Enterococcus faecalis* (3.10), *L. lactis* (2.80), and *Ligilactobacillus ruminis* (2.68). Young adult microbiomes were dominated by *S. gallolyticus* (4.05), with concurrent enrichment of cellulose-degrading taxa including *Clostridium isatidis* (3.01) and *Ruminococcus* sp. SR1/5 (2.47). Mature adult microbial signatures comprised *Escherichia ruysiae* (3.63) and *Clostridium perfringens* (3.38), alongside bacteriophage enrichment including *Escherichia* phage vB_EcoP-ZQ2 (2.67) and *Gamaleyavirus* Sb1 (2.54). Advanced adults demonstrated *E. coli* predominance (5.48), with opportunistic pathogens *Klebsiella pneumoniae* (3.84) and *Shigella flexneri* (3.83) notably enriched. Longevity adults displayed the most diversified microbial community composition, characterized by *A. caviae* (4.10), *P. fluorescens* (4.06), *Veillonella parvula* (3.95), *Hafnia alvei* (3.75), and *R. gnavus* (3.33) (Figure 2C).

### Age-dependent distribution patterns of longevity-associated probiotic species in giant pandas

CLR-transformed relative abundance analysis revealed distinct age-dependent enrichment patterns of 4 potential longevity-associated bacterial species across giant panda life stages, with particularly pronounced enrichment in longevity adults (LA).

*R. gnavus* demonstrated significant enrichment in LA cohorts (*P* < 0.05; mean CLR = 2.18), substantially exceeding values observed in mature (0.85), advanced (0.45), young (0.32), prime (0.27), and adolescent (0.08) adults. The LA group was characterized by considerable inter-individual variation (CLR range: −0.30 to 8.70), suggesting heterogeneous microbial compositional profiles within longevity populations (Figure 3A).

**Figure 3:**
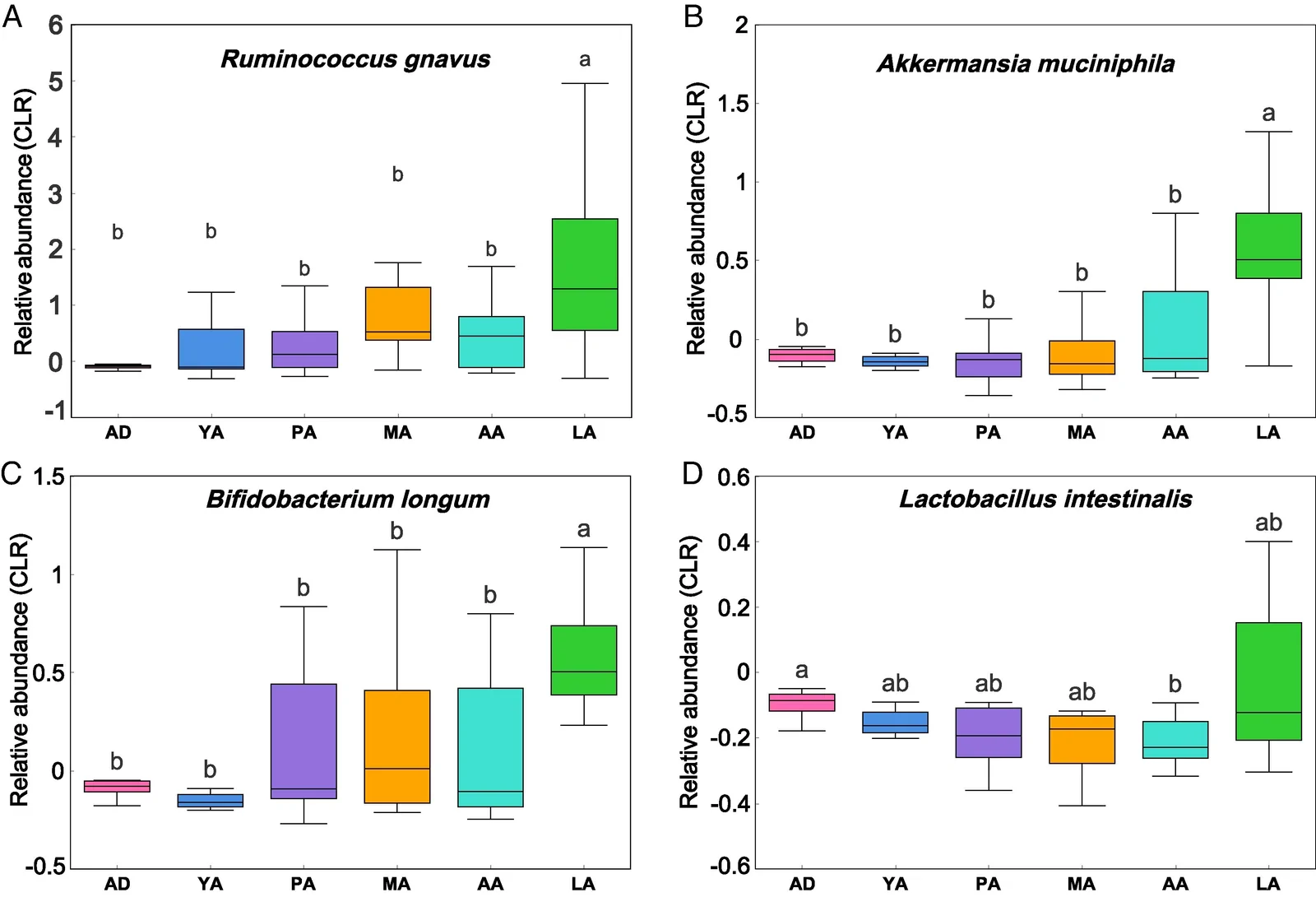
Age-dependent distribution patterns of longevity-associated probiotic species in captive giant pandas. (A-D) Distribution of *R. gnavus*, *A. muciniphila*, *B. longum*, and *L. intestinalis*, respectively. Relative abundance shown as CLR-transformed values across 6 age stages. Different letters indicate significant differences (*P* < 0.05). AD: adolescent; YA: young adult; PA: prime Adult; MA: mature adult; AA: advanced adult; LA: longevity adult.

*A. muciniphila* exhibited comparable enrichment patterns in LA giant pandas (*P* < 0.05; mean CLR = 0.62), surpassing advanced (0.08), adolescent (−0.002), mature (−0.0004), prime (−0.08), and young (−0.11) cohorts (Figure 3B).

*B. longum* followed similar distribution patterns with significant LA enrichment (*P* < 0.05; mean CLR = 0.68) relative to adolescent (0.18), mature (0.17), prime (0.12), advanced (0.11), and young (−0.16) groups. Notably, elevated abundances in select adolescent individuals (maximum CLR = 2.69) indicated potentially different ecological functions across developmental stages (Figure 3C).

*L. intestinalis* displayed distinct non-linear distribution dynamics. Relatively higher abundances characterized adolescents (mean CLR = −0.02), with significant depletion in advanced adults (mean CLR = −0.21; *P* < 0.05), followed by recovery in LA cohorts (mean CLR = −0.07). Multiple comparison analysis confirmed significant differences between adolescent and advanced adult groups (*P* < 0.05), whereas LA comparisons with both groups remained non-significant (Figure 3D).

### Enterotype succession across life stages

DMM clustering of genus-level abundance data from 82 age-stratified fecal samples revealed optimal partitioning into 4 distinct enterotypes: E1 (*n* = 18), E2 (*n* = 19), E3 (*n* = 27), and E4 (*n* = 18). Enterotype-driving genera comprised *Escherichia* and *Cellulosilyticum* (E1), *Streptomyces* and *Turicibacter* (E2), *Streptococcus* and *Enterococcus* (E3), and *Salmonella* and *Shigella* (E4) (Figure 4A, C, D, Supplementary Figure S2).

**Figure 4:**
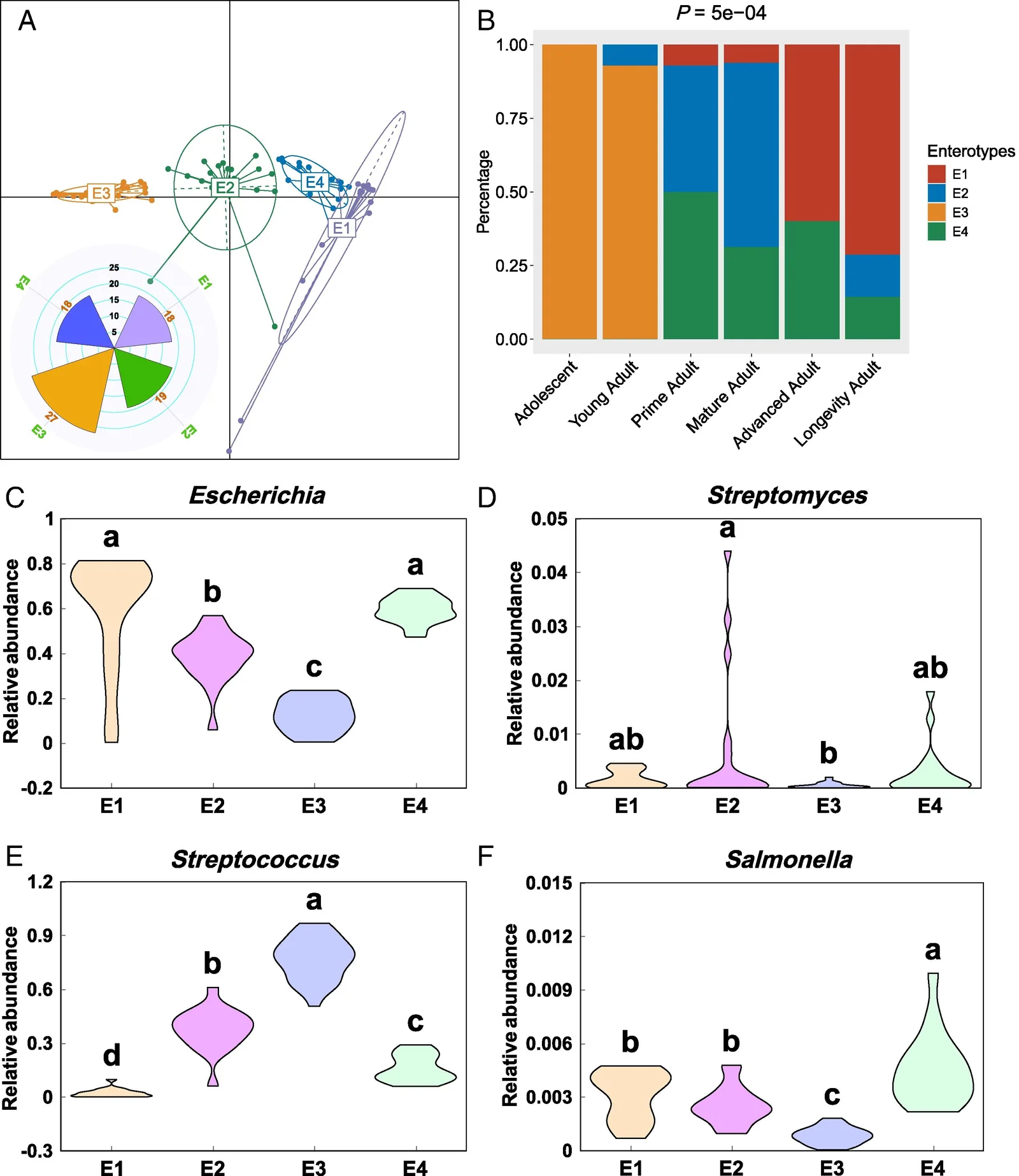
Enterotype succession and characteristic genera distribution across giant panda life stages. (A) DMM clustering visualization showing 4 distinct enterotypes. (B) Age-dependent enterotype distribution revealing significant transitions. (C–F) Relative abundance of enterotype-driving genera: *Escherichia* (C), *Streptomyces* (D), *Streptococcus* (E), and *Salmonella* (F) across the 4 enterotypes. Different letters indicate significant differences (*P* < 0.05).

Highly significant age-dependent enterotype transitions were observed (*P* = 5 × 10^−4^). E3 predominated in adolescent and young adult cohorts (≥92.86%), reflecting early life-stage dominance of LAB. Prime and mature adults demonstrated increased E2 (42.86% and 62.5%, respectively) and E4 (50% and 31.5%, respectively) prevalence. Advanced adults exhibited exclusive E1 and E4 distribution (100% combined), indicating *Escherichia* and *Salmonella* predominance in senescence (Figure 4B).

Longevity adults displayed a distinctive enterotype profile characterized by E1 dominance (71.43%), paralleling advanced adults in this respect. However, they uniquely retained E2 (14.29%) and E4 (14.29%) representation. This hybrid distribution pattern, incorporating both early-life and senescent features, potentially reflects adaptive microbiome regulatory mechanisms underlying longevity in giant pandas.

### Core metabolic pathway abundance patterns

PCoA of functional profiles revealed pronounced age-related clustering in giant panda gut microbiomes. For MetaCyc pathway profiles (Figure 5A), PCoA1 explained 81.4% of total variance and significantly separated age groups (PERMANOVA *R*^2^ = 0.67, *P* = 0.001). Adolescent giant pandas were positioned at the positive end of this axis, whereas older cohorts were distributed progressively toward the negative side. Longevity adults exhibited broad dispersion along PCoA2 (5.8% variance). CAZymes-based PCoA (Figure 5B) captured substantial variance proportions (PCoA1: 63.1%; PCoA2: 19.9%) and revealed pronounced age-dependent stratification (*R*^2^ = 0.24, *P* = 0.001), with distinct clustering of age groups along the primary axis.

**Figure 5:**
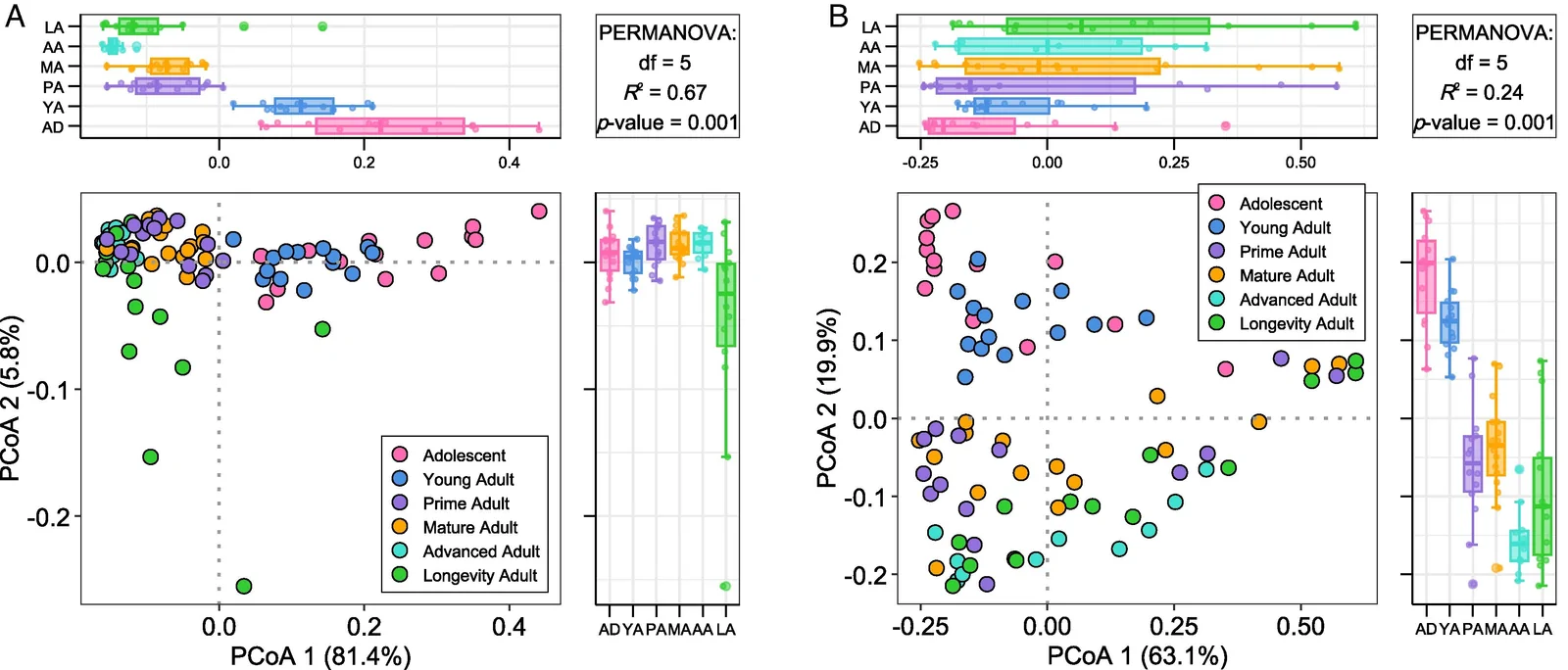
Age-related clustering patterns of gut microbiome functional profiles in captive giant pandas. (A, B) PCoA of gut microbiome functional profiles across giant panda age groups based on MetaCyc metabolic pathways (A) and CAZyme family annotations (B). AD: adolescent; YA: young adult; PA: prime adult; MA: mature adult; AA: advanced adult; LA: longevity adult.

MetaCyc database annotation (Figure 6A) revealed differential distributions of the most abundant metabolic pathways in the giant panda gut microbiome, underscoring pivotal roles in physiological regulation throughout aging. Consistently high abundance was observed for the cell wall synthesis-related peptidoglycan maturation pathway (PWY0-1586) in all age groups, with relative levels rising from adolescents (0.0124 ± 0.0007%) to advanced adults (0.0159 ± 0.0005%). Gradual declines were detected in amino acid biosynthesis (VALSYN-PWY, ILEUSYN-PWY) and fermentation (PWY-7111) pathways, exemplified by the decrease in VALSYN-PWY from 0.0127 ± 0.0026% among adolescents to 0.0079 ± 0.0013% in longevity adults. Carbohydrate metabolism exhibited distinct patterns: the nonoxidative branch of the pentose phosphate pathway (NONOXIPENT-PWY) maintained stable abundance. In contrast, the lactose degradation pathway (LACTOSECAT-PWY) underwent pronounced age-dependent reduction from 0.0162 ± 0.0057% in adolescents to 0.0012 ± 0.0007% in advanced adults. Dynamic shifts in these high-abundance pathways may reflect critical adaptations in energy metabolism and nutritional demands at different developmental stages.

**Figure 6:**
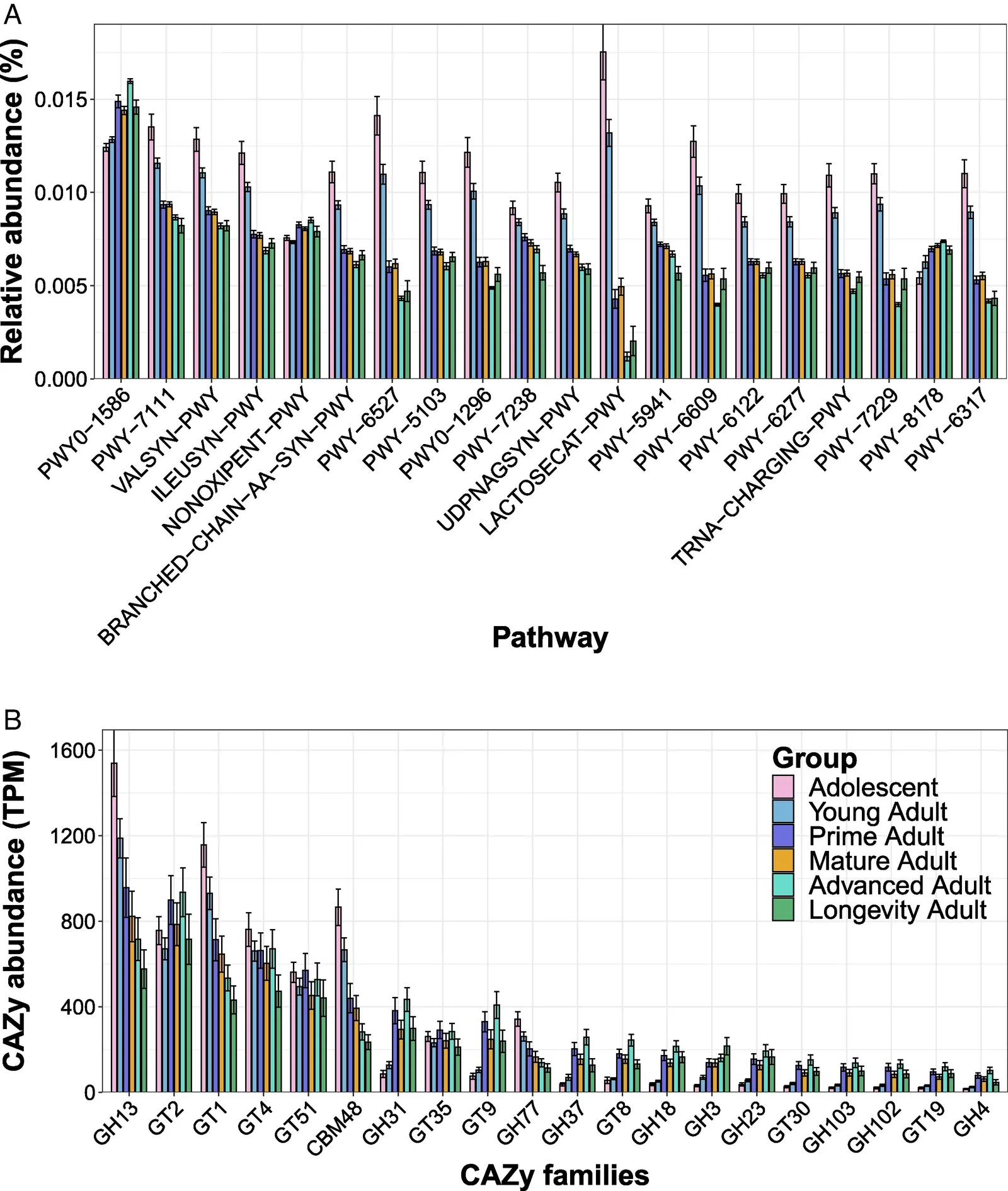
Age-dependent distribution of the top 20 most abundant metabolic pathways in giant panda gut microbiomes. (A) MetaCyc metabolic pathway relative abundance across 6 age groups. (B) CAZyme family abundance (TPM) across 6 age groups.

Functional annotation of CAZymes (Figure 6B) revealed age-dependent distribution patterns in the giant panda gut microbiome, reflecting carbohydrate-active enzyme roles in dietary fiber degradation and metabolic regulation. The GH13 family (α-amylase and related enzymes) predominated across all age groups, maintaining high abundance from adolescents (1549 ± 582 TPM) through advanced adults (1,413 ± 100 TPM) before declining in longevity adults (611 ± 309 TPM). Glycosyltransferase families displayed distinct developmental trajectories. GT2, involved in cell wall polysaccharide synthesis, remained relatively stable through young adults (726 ± 186 TPM in adolescents; 708 ± 166 TPM in young adults). Abundance then increased in prime and mature adults, ultimately peaking in advanced adults (1,240 ± 177 TPM). In contrast, GT1 showed the highest abundance in adolescents (1,177 ± 339 TPM), followed by progressive decline toward longevity adults (491 ± 198 TPM), suggesting shifting biosynthetic demands across life stages. Carbohydrate-binding modules demonstrated pronounced age-related changes. CBM48, containing cellulose-binding domains, decreased markedly from adolescents (742 ± 312 TPM) to longevity adults (233 ± 93 TPM), suggesting reduced cellulolytic capacity in aged individuals. Families mediating complex carbohydrate modification increased in prime and mature adults: GT35 (phosphorylase family) and GH31 (α-glucosidase family) reached peak abundances in advanced adults (393 ± 35 TPM and 445 ± 43 TPM, respectively). GT51 (murein biosynthesis) and GT4 (sucrose synthase family) maintained stable abundance throughout development, suggesting their consistent roles in cell wall maintenance and energy metabolism. These patterns demonstrate a functional transition across the lifespan. Glycoside hydrolases for fiber degradation and glycosyltransferases for biosynthesis dominate in adolescents. Carbohydrate modification and structural metabolism predominate in adulthood, whereas overall carbohydrate-processing capacity declines in longevity adults.

### Age-specific metabolic pathway biomarkers

Age-dependent functional profiling identified 74 significantly different metabolic pathways and 8 CAZyme families (LDA > 2, *P* < 0.05) in Adolescent giant pandas. Young Adults showed 14 MetaCyc pathways and 4 CAZyme families, whereas prime adults and mature adults exhibited 11 and 12 MetaCyc pathways, respectively. Advanced adults demonstrated the greatest functional enrichment (156 MetaCyc pathways, 25 CAZyme families), and longevity adults maintained high diversity (71 MetaCyc pathways, 25 CAZyme families) (Figure 7A, B).

**Figure 7:**
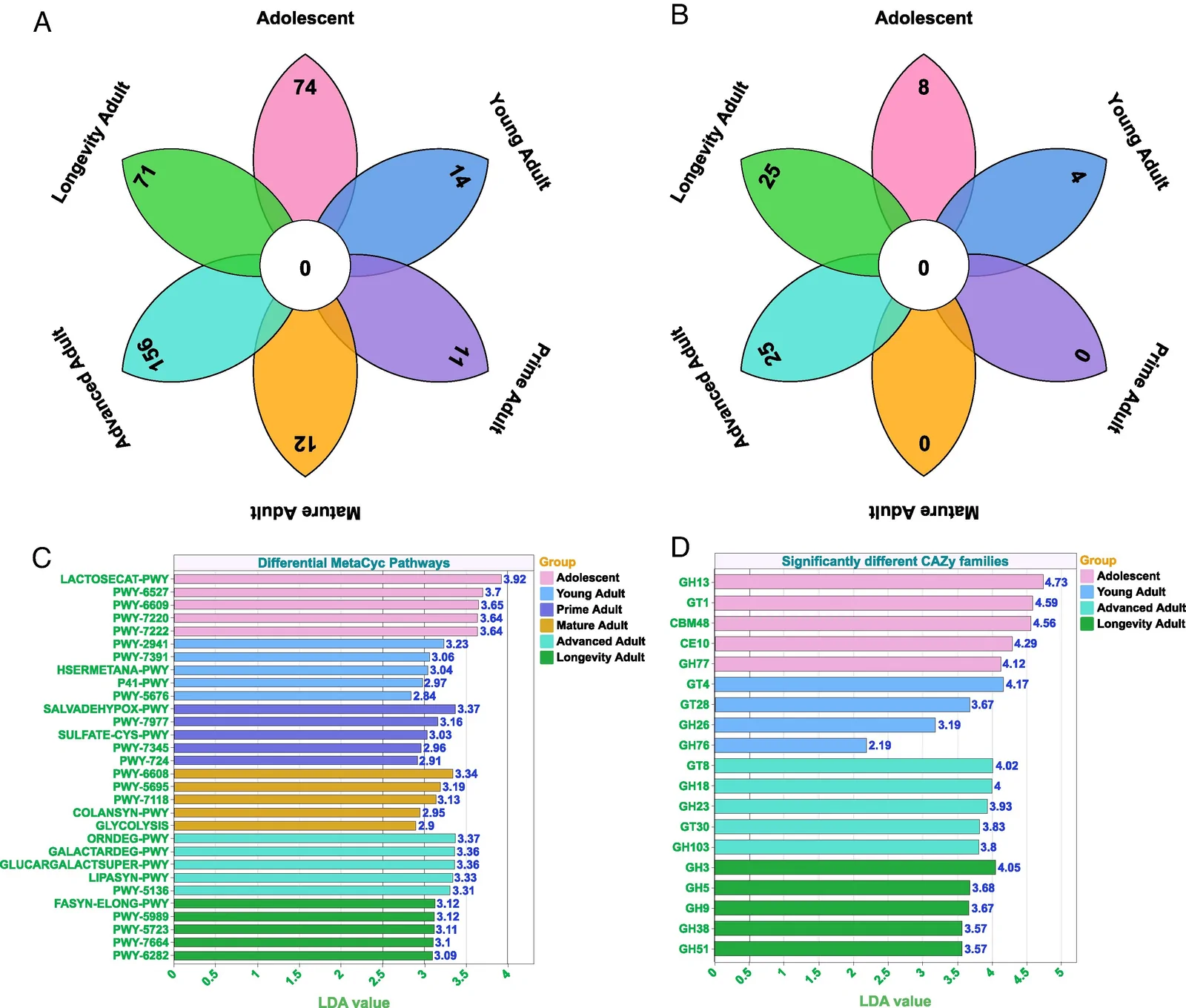
Age-specific metabolic pathway biomarkers identified by LEfSe analysis in giant panda gut microbiomes. (A, B) Venn diagrams showing differentially abundant pathways across 6 age groups for MetaCyc metabolic pathways (A) and CAZyme families (B). (C, D) LDA scores of top differential pathways colored by age group for MetaCyc annotations (C) and CAZyme family annotations (D).

Differential metabolic pathway enrichment across age cohorts revealed distinct functional signatures. Adolescent giant pandas exhibited predominant enrichment in carbohydrate degradation and nucleotide metabolism: LACTOSECAT-PWY (lactose and galactose degradation I; LDA = 3.92), PWY-6527 (stachyose degradation; 3.70), PWY-6609 (adenine and adenosine salvage III; 3.65), PWY-7220 (adenosine deoxyribonucleotides de novo biosynthesis II; 3.64), and PWY-7222 (guanosine deoxyribonucleotides de novo biosynthesis II; 3.64). Amino acid biosynthesis pathways characterized young adults: PWY-2941 (L-lysine biosynthesis II; 3.23), PWY-7391 (isoprene biosynthesis II engineered; 3.06), and HSERMETANA-PWY (L-methionine biosynthesis III; 3.04). Prime adults displayed enrichment in purine salvage and sulfate assimilation: SALVADEHYPOX-PWY (adenosine nucleotides degradation II; 3.37), PWY-7977 (L-methionine biosynthesis IV; 3.16), and SULFATE-CYS-PWY (superpathway of sulfate assimilation and cysteine biosynthesis; 3.03). Mature adults featured purine degradation and complex carbohydrate metabolism: PWY-6608 (guanosine nucleotides degradation III; 3.34), PWY-5695 (inosine 5′-phosphate degradation; 3.19), and PWY-7118 (chitin deacetylation; 3.03). Advanced adults demonstrated enrichment in amino acid degradation and lipid biosynthesis: ORNDEG-PWY (superpathway of ornithine degradation; 3.37), GALACTARDEG-PWY (D-galactarate degradation I; 3.36), GLUCARGALACTSUPER-PWY (superpathway of D-glucarate and D-galactarate degradation; 3.36), NAGLIPASYN-PWY (lipid IVA biosynthesis; 3.33), and PWY-5136 (fatty acid β-oxidation II; 3.31). Longevity adults exhibited predominant lipid metabolism, particularly fatty acid elongation: FASYN-ELONG-PWY (fatty acid elongation-saturated; 3.12), PWY-5989 (stearate biosynthesis II; 3.12), PWY-5723 (Rubisco shunt; 3.11), PWY-7664 (oleate biosynthesis IV anaerobic; 3.10), and PWY-6282 (palmitoleate biosynthesis I; 3.09) (Figure 7C).

Differential CAZyme family abundance analysis revealed age-dependent carbohydrate metabolism specialization. Adolescent giant pandas demonstrated the highest enrichment in starch-degrading enzymes, with GH13 (LDA = 4.73), GT1 (4.59), and carbohydrate-binding module CBM48 (4.56) predominating, accompanied by CE10 (4.29) and GH77 (4.12). Young adults exhibited enrichment in glycan biosynthesis-related families GT4 (4.17) and GT28 (3.67), along with β-mannanase GH26 (3.19) and α-mannosidase GH76 (2.19). Advanced adults displayed significant enrichment in glycosyltransferase GT8 (4.02), chitinase GH18 (4.00), peptidoglycan lytic transglycosylases GH23 (3.93) and GH103 (3.80), and GT30 (3.83). Longevity adults uniquely featured cellulolytic enzyme enrichment, with β-glucosidase GH3 (4.05), endoglucanase GH5 (3.68), and cellulose-degrading GH9 (3.67), alongside α-mannosidase GH38 (3.57) and α-L-arabinofuranosidase GH51 (3.57) showing pronounced enrichment (Figure 7D).

### Age-dependent expansion of BGCs in the giant panda gut microbiome

To characterize secondary metabolite biosynthetic capacity, we performed comprehensive BGC profiling using antiSMASH on MEGAHIT-assembled metagenomic contigs. For BGC compositional profiles (Figure 8A), PCoA1 explained 51.4% of total variance and significantly separated age groups (*R*^2^ = 0.26, *P* = 0.001). Adolescent pandas were positioned at the positive end of this axis, whereas older cohorts were distributed progressively toward the negative side. Longevity adults exhibited broad dispersion along PCoA2 (12.8% variance).

**Figure 8:**
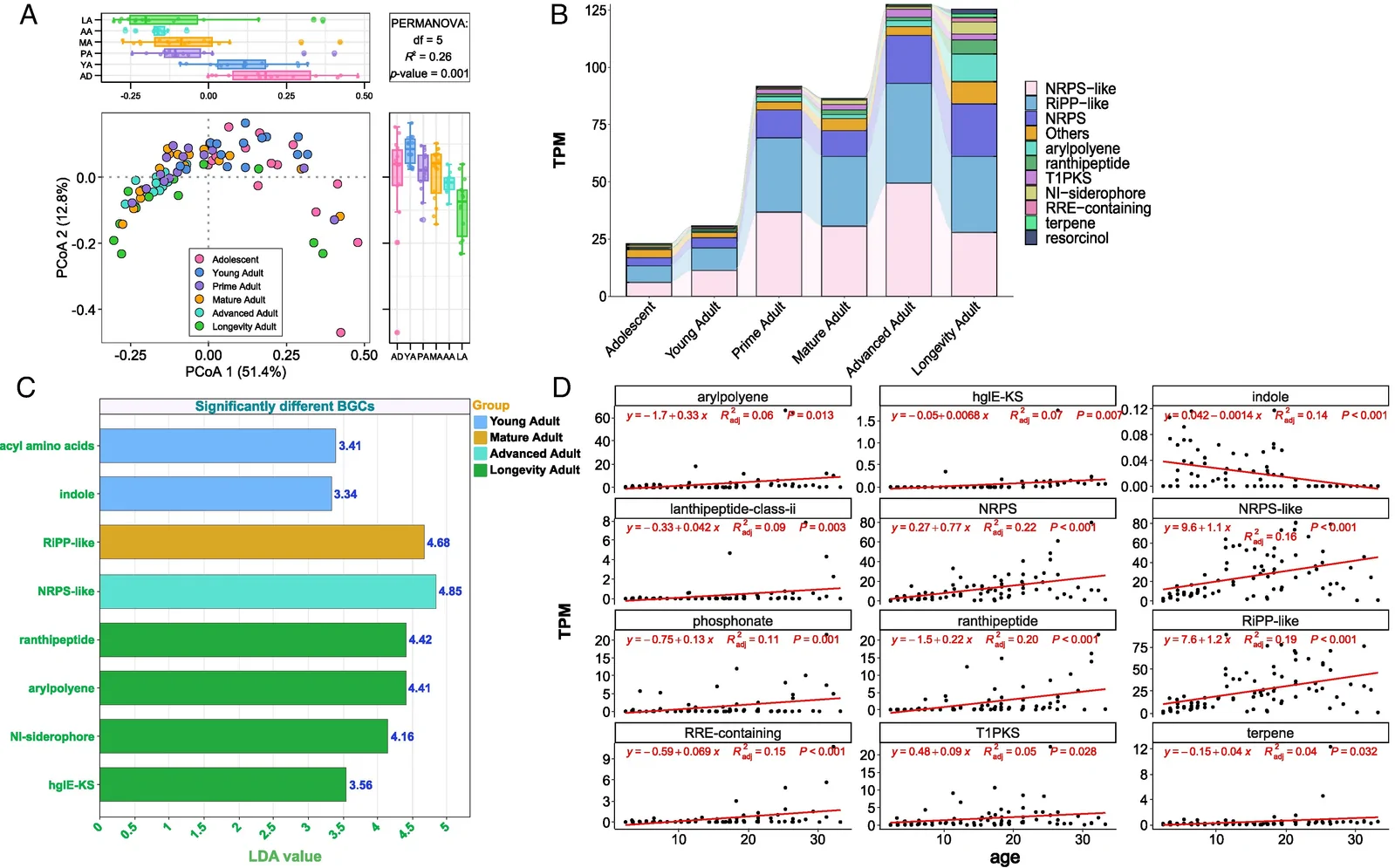
Age-dependent expansion of biosynthetic gene clusters in the giant panda gut microbiome. (A) PCoA of BGC compositional profiles showing age-dependent clustering patterns. PERMANOVA statistics and box plots display coordinate distributions across age groups. (B) Stacked bar charts illustrating total BGC abundance and compositional distribution across 6 life stages. (C) LEfSe analysis revealing age-specific BGC enrichment signatures, with bar length representing LDA score magnitude (LDA > 2 and *P* < 0.05). (D) Linear regression analyses of BGC families demonstrating significant age correlations, with regression statistics displayed for each BGC type.

Total BGC abundance increased approximately 5-fold across the lifespan, from 22.7 TPM in Adolescents to 125.2 TPM in longevity adults, with peak levels (127.5 TPM) in advanced adults (Figure 8B). NRPS (non-ribosomal peptide synthetase) and NRPS-like pathways constituted the predominant biosynthetic systems, exhibiting marked expansion from adolescents to advanced adults (approximately 6-fold and 8-fold increases, respectively). RiPP-like (ribosomally synthesized and post-translationally modified peptides) pathways demonstrated parallel accumulation, increasing approximately 6-fold from adolescents to advanced adults before moderately declining in longevity adults. Arylpolyene biosynthesis exhibited pronounced expansion in longevity adults, with an approximately 21-fold increase relative to adolescents. Terpene, T1PKS (type I polyketide synthase), and NI-siderophore pathways displayed modest but significant age-dependent increases.

LDA revealed distinct biosynthetic signatures across life stages (LDA > 2, *P* < 0.05; Figure 8C). Young adults exhibited enrichment in acyl amino acids and indole derivatives. Mature adults demonstrated enrichment in RiPP-like pathways associated with antimicrobial peptide production. Advanced adults displayed distinctive enrichment of NRPS-like systems, reflecting intensified non-ribosomal peptide biosynthesis. Longevity adults exhibited the greatest diversity of enriched BGC types, including ranthipeptides, arylpolyene, NI-siderophore, and hglE-KS systems, indicating preserved biosynthetic complexity in long-lived individuals.

Linear regression analysis identified significant age correlations for 12 of 24 examined BGC families (*P* < 0.05), with 11 showing positive and one showing negative associations (Figure 8D). NRPS pathways exhibited the strongest positive age association (*R*^2^ = 0.22, *P* < 0.001), increasing from 3.6 TPM in adolescents to 23.1 TPM in longevity adults. Ranthipeptide biosynthesis showed the second strongest correlation (*R*^2^ = 0.20, *P* < 0.001), increasing dramatically across the lifespan. RiPP-like and NRPS-like pathways displayed similarly robust positive age associations (*R*^2^ = 0.19 and 0.16, respectively; both *P* < 0.001). Conversely, indole biosynthesis exhibited significant negative age correlation (*R*^2^ = 0.14, *P* < 0.001), representing the only BGC type to decline with age, suggesting metabolic remodeling of tryptophan utilization pathways.

### Age-associated restructuring of taxonomic and functional profiles transcends environmental confounders

Univariate PERMANOVA revealed that life stage explained the greatest variance in both genus-level composition (*R*^2^ = 0.616) and pathway profiles (*R*^2^ = 0.674), substantially exceeding location effects (*R*^2^ = 0.215–0.249). Sex showed no significant association (*R*^2^ < 0.01), and birth origin exhibited weak effects (Supplementary Table S3). When simultaneously accounting for all confounders in multivariate PERMANOVA, life stage remained the predominant factor. At the genus level, life stage independently explained 38.2% of variance (pseudo-F = 15.8, *P* < 0.001), whereas other variables contributed minimally (location *R*^2^ = 0.023, *P* = 0.146; sex *R*^2^ = 0.003, *P* = 0.503; birth origin *R*^2^ = 0.015, *P* = 0.043). At the functional level, life stage accounted for 47.7% of pathway variation whereas all other factors were non-significant (all *P* > 0.15).

MaAsLin3 multivariate modeling, adjusting for sex, location, and birth origin, identified 310 genera with significant age-associated abundance changes (*P* < 0.05). Of these, 31 genera decreased with age, most prominently *Streptococcus* (*β* = −1.89), *Leuconostoc* (−1.33), and *Agathobacter* (−1.27). In contrast, 279 genera increased, including *Aeromonas* (1.81), *Pseudomonas* (1.40), and *Acinetobacter* (1.29) (Supplementary Table S4). At the metabolic pathway level, MaAsLin3 identified 200 MetaCyc pathways with significant age-related changes (*P* < 0.05). Ninety-four pathways decreased with age, such as UDPNACETYLGALSYN-PWY (UDP-N-acetyl-D-galactosamine biosynthesis; −1.97) and LACTOSECAT-PWY (lactose degradation; −1.42), whereas 106 pathways increased, including HEME-BIOSYNTHESIS-II (0.86) and GLUCUROCAT-PWY (0.93) (Supplementary Table S5).

To validate these findings, we compared MaAsLin3 results with prior LEfSe/Kruskal-Wallis analyses. Among the 97 genera and 338 pathways identified by LEfSe, 37 genera (38%) and 179 pathways (53%) overlapped with MaAsLin3 markers. Key age-associated signatures—including depleted *Streptococcus*, *Leuconostoc*, and LACTOSECAT-PWY, as well as enriched *Escherichia*, *Aeromonas*, and HEMESYN2-PWY—were consistent across both approaches (Supplementary Table S6).

### Sex-associated microbial and functional biomarkers

Although sex showed no significant effect on overall microbiome composition (*R*² < 0.01, *P* = 0.503, Supplementary Table S3), we performed sex-stratified LEfSe analysis. Sex represents a fundamental biological variable influencing gut microbiota across mammalian species. We examined taxonomic abundance at genus and species levels, MetaCyc pathway representation, and CAZyme family distribution. Using standard thresholds (LDA > 2.0, *P* < 0.05), we identified 10 sex-associated biomarkers spanning taxonomic, metabolic, and enzymatic features.

At the genus level, 4 bacterial taxa were significantly enriched in females: *Flavobacterium* (LDA = 2.86), *Plesiomonas* (2.65), *Helicobacter* (2.53), and *Paenibacillus* (2.08). No genera were significantly enriched in males.

Functional profiling revealed sex-specific enrichment of 4 metabolic pathways. Females showed elevated abundance of colanic acid biosynthesis (COLANSYN-PWY; 2.24) and glycolysis (2.08). In contrast, males exhibited enrichment of superpathway PWY-7211 (2.30) and L-threonine biosynthesis (THRESYN-PWY; 2.06).

CAZyme analysis identified 2 families enriched in females: glycoside hydrolase GH101 (2.86) and glycosyltransferase GT6 (2.48). These findings demonstrate that although sex does not shape overall microbiome architecture, it influences specific taxonomic and functional features of the giant panda gut microbiome (Supplementary Table S7).

### Isolation and species identification of LAB

Gradient dilution and strict anaerobic cultivation on calcium carbonate-supplemented MRS agar yielded 98 LAB isolates from giant panda fecal samples. Molecular identification via 16S rRNA gene sequencing classified these strains into 8 species: *Pediococcus acidilactici* (35 isolates), *Enterococcus gallinarum* (1), *Enterococcus faecium* (2), *Ligilactobacillus murinus* (13), *L. intestinalis* (21), *Lactobacillus johnsonii* (24), *Limosilactobacillus reuteri* (1), and *Limosilactobacillus balticus* (1) (Figure 9A, B).

**Figure 9:**
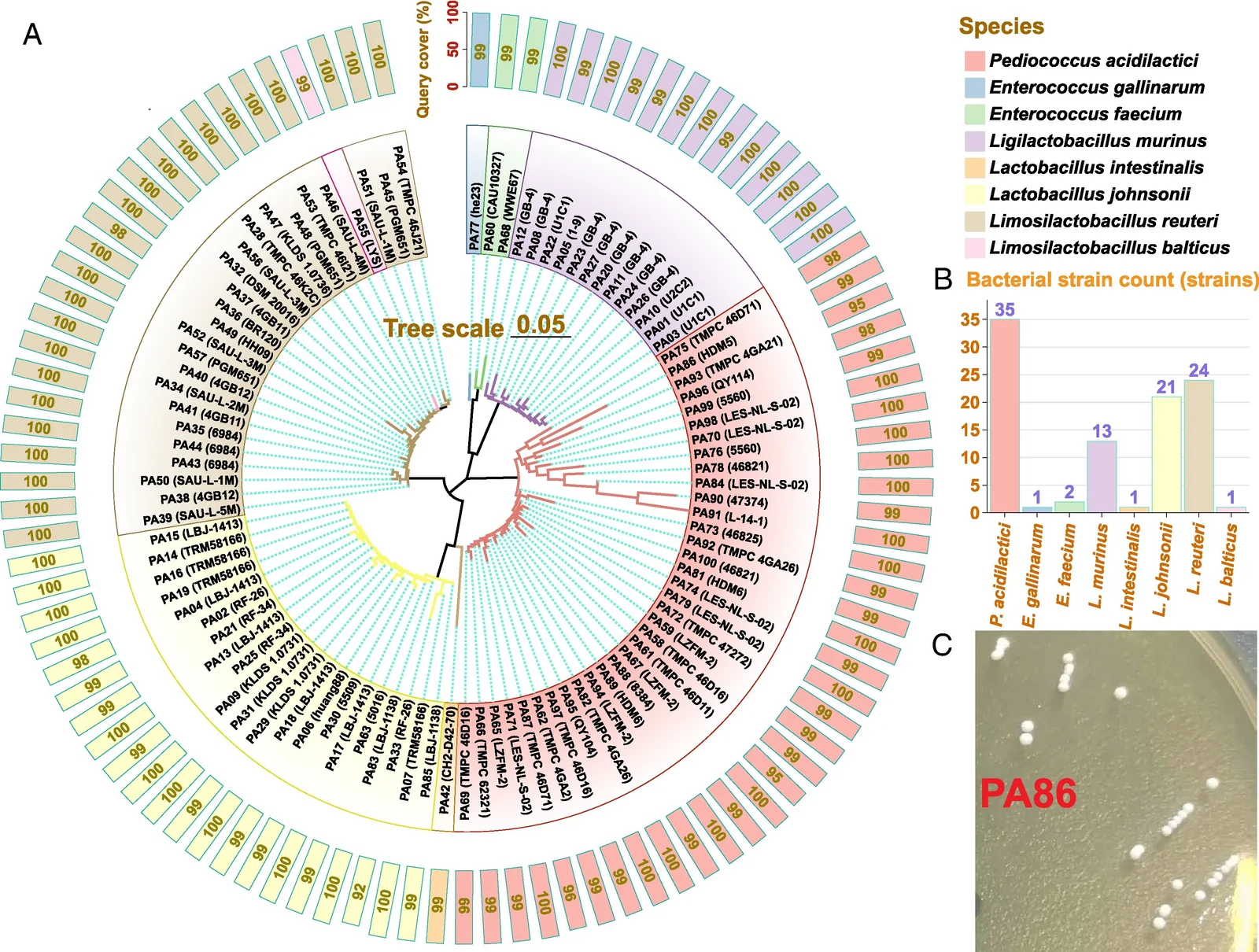
Isolation and identification of LAB from giant panda fecal samples. (A) The colored bars in the outer ring indicate query coverage percentages from BLAST alignment. Isolate designations are shown in the inner ring, with parenthetical annotations indicating the species with the highest sequence similarity. (B) Species distribution of isolated strains. (C) Colony morphology of *P. acidilactici* strain PA86 on MRS agar.

Morphological characterization of the dominant species *P. acidilactici* (strain PA86) revealed circular, smooth colonies with cream to pale yellow pigmentation, regular margins, and a moist, glossy surface on MRS agar (Figure 9C).

Phylogenetic reconstruction based on 16S rRNA sequences delineated evolutionary relationships and quantitative distribution patterns among isolates. Query coverage ranging from 92% to 100% confirmed identification reliability (Figure 9A). Significant interspecific genetic distances contrasted with minimal intraspecific variation, providing a robust framework for investigating population structure and functional diversity of giant panda intestinal LAB.

### Safety assessment of bacterial isolates

Hemolytic activity assessment revealed that all 98 bacterial isolates exhibited γ-hemolysis (non-hemolytic phenotype) on sheep blood agar plates, as demonstrated by the absence of clearing zones around bacterial colonies. *S. aureus* (SA) served as a positive control, displaying characteristic hemolysis (Figure 10A, B).

**Figure 10:**
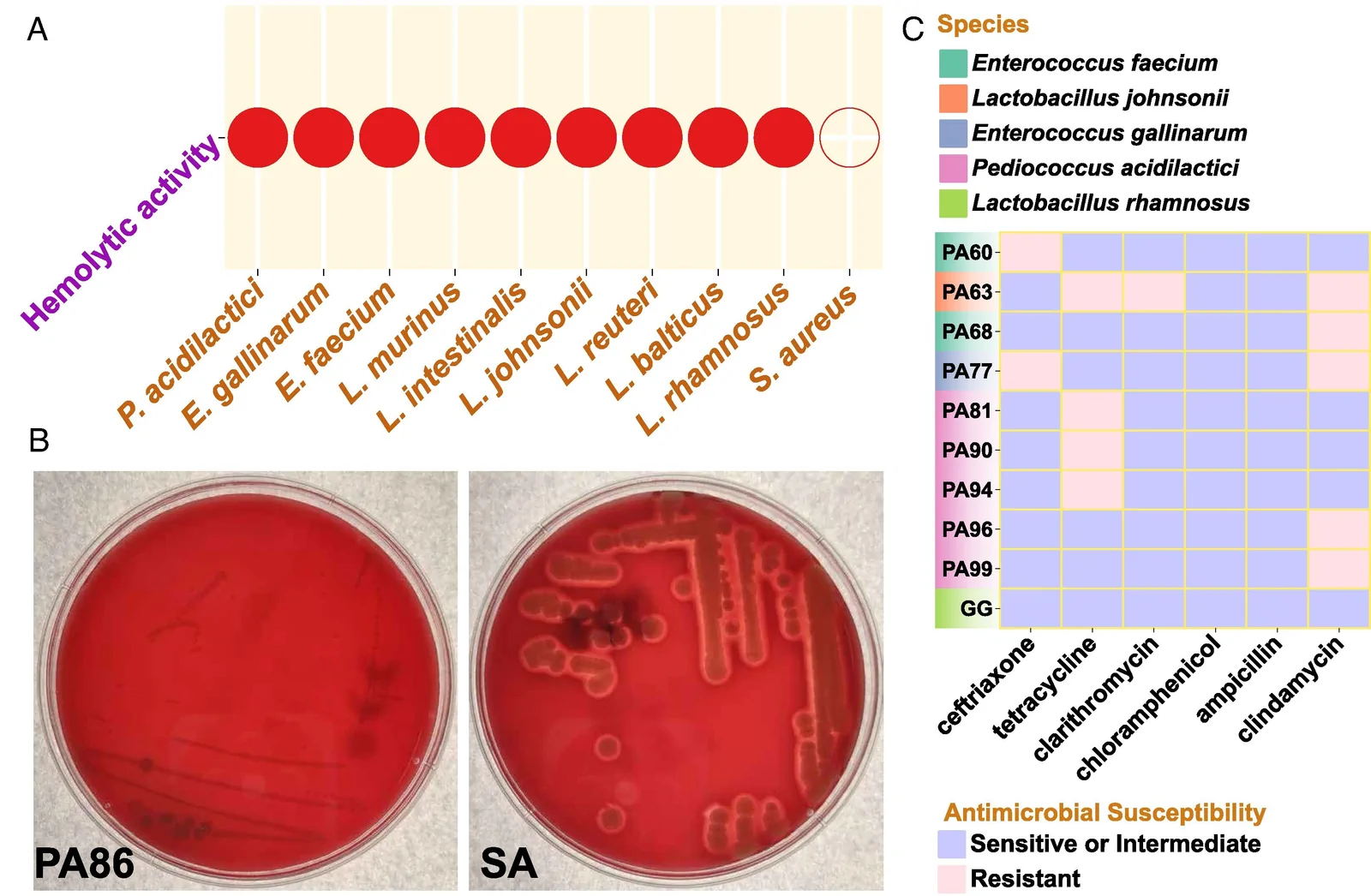
Hemolytic activity and antimicrobial susceptibility profiles of bacterial isolates. (A) Hemolytic activity screening of 98 bacterial isolates on sheep blood agar plates, with all strains demonstrating γ-hemolysis (non-hemolytic phenotype). (B) Representative images of non-hemolytic strain PA86 and *S. aureus* (SA) control on blood agar plates. (C) Antimicrobial resistance patterns of selected bacterial isolates against 6 antibiotics, with LGG as reference strain.

Antimicrobial susceptibility testing against 6 commonly prescribed antibiotics (ceftriaxone, tetracycline, clarithromycin, chloramphenicol, ampicillin, and clindamycin) was performed using the disk diffusion method. LGG was employed as a probiotic reference strain. Among the 98 isolates tested, 89 strains (90.81%) demonstrated susceptibility to all antibiotics examined. However, 9 strains (9.2%) exhibited varying degrees of resistance to one or more antibiotics (Figure 10C).

Notably, antimicrobial resistance was predominantly observed among *Enterococcus species. E. faecium* and *E. gallinarum* isolates displayed resistance patterns to multiple antibiotics, including tetracycline, clarithromycin, and clindamycin. *Lactobacillus* and *Pediococcus* isolates generally maintained susceptibility profiles comparable to the reference strain. In accordance with established safety criteria for probiotic selection, all strains exhibiting antimicrobial resistance were excluded from further characterization.

## Discussion

Aging in the giant panda, a long-lived non-human species, was investigated to address the critical gap in mammalian gut microbiome research, which has remained predominantly human-centric (Huang et al. 2023). Machine learning-driven enterotype stratification, functional metagenomic profiling, and cross-species comparative analysis were employed to identify distinct longevity-associated microbial taxa and metabolic pathways.

Enterotypes represent distinct gut microbial community structures shaped by diet, host sex, antibiotic exposure, and age (Wu et al. 2011; Zhang et al. 2021; Lee et al. 2023). In humans, centenarians exhibit youthful microbial signatures, including *Bacteroides*-dominated enterotypes, elevated species diversity, enrichment of beneficial commensals, and depletion of pathobionts. This pattern contrasts with typical age-related decline (Pang et al. 2023). To examine whether similar patterns—wherein long-lived individuals retain youthful microbiota characteristics—extend to other mammals, we applied DMM clustering to giant panda gut microbiomes across life stages. Age-specific enterotypes emerged, with young and middle-aged pandas harboring distinct community structures. Notably, the longest-lived elderly pandas displayed a *Streptomyces*-enriched enterotype more similar to that of young individuals than to other elderly pandas without extended longevity. *Streptomyces* is particularly noteworthy, as this genus—whereas predominantly associated with soil environments—can also establish residence in mammalian gastrointestinal tracts. Gut-adapted *Streptomyces* strains exhibit functional properties including antimicrobial activity and gastrointestinal tolerance (Bolourian and Mojtahedi 2018; Cuozzo et al. 2023; Verma et al. 2024). These properties suggest potential ecological roles beyond their terrestrial origins. However, we emphasize several important caveats. First, longevity in captive pandas occurs under managed care conditions that may influence both lifespan and microbiota composition independently. Second, we lack physiological or clinical data (e.g., metabolic markers, disease status) to establish whether these youthful microbiota profiles confer actual health benefits. Third, these metagenomic observations represent correlative associations rather than causal relationships. Experimental validation is required to determine whether specific microbial taxa directly contribute to extended lifespan or merely reflect it.

### Probiotic enrichment in longevity-associated microbiomes

Significant reorganization of the gut microbiome was observed in long-lived elderly giant pandas, characterized by markedly increased microbial diversity compared with younger individuals and enrichment of multiple recognized probiotic species. Such findings align closely with the microbial profiles of healthy aging populations in humans and other mammals. Mammalian aging is typically accompanied by dysbiosis characterized by decreased gut microbial diversity and increased pathogenic bacteria. Healthy long-lived individuals, however, frequently deviate from this pattern (Ghosh et al. 2022). Gut microbial diversity in Chinese centenarians shows an increasing trend with advanced age, with longevity-associated microbiomes enriched in anti-inflammatory and short-chain fatty acid (SCFA)-producing commensals (Wu et al. 2022). Long-lived giant pandas exhibited similar characteristics: significant enrichment of species potentially promoting host metabolic and immune health, including *A. muciniphila*, *B. longum*, *L. intestinalis*, and moderate levels of *R. gnavus*. Such enrichment may establish a microecological balance incorporating advantageous features of both young and elderly microbiomes.

*A. muciniphila*, significantly enriched in long-lived individuals, plays crucial roles in host intestinal barrier function and metabolic homeostasis. Specifically colonizing the intestinal mucus layer, this bacterium derives energy through mucin glycoprotein degradation, with metabolites subsequently serving as carbon sources for other commensals, establishing mutualistic ecological networks (Reunanen et al. 2015). *A. muciniphila* stimulates mucus secretion by intestinal epithelial cells and enhances tight junctions, thereby strengthening mucosal integrity and reducing pathogen and toxin translocation to alleviate inflammaging (Cani et al. 2022). Notably, terpene BGCs show significant enrichment in super-centenarian gut microbiomes, with BGC abundance positively correlating with *A. muciniphila* distribution. This correlation further supports the bacterium's potential mechanistic role in modulating longevity-associated metabolic pathways (Liu et al. 2024).

*B. longum*, a prevalent probiotic in human and animal intestinal tracts, demonstrates increased relative abundance in long-lived elderly individuals and exerts multiple beneficial mechanisms (Xiao et al. 2021). *B. longum* enhances intestinal mucosal immunity by promoting secretory antibody production, particularly IgA, which establishes an immune barrier within the intestinal lumen. Significant increases in beneficial SCFA-producing bacteria accompany these effects (Kurata et al. 2022). Anti-inflammatory properties of *B. longum* are evidenced by downregulation of pro-inflammatory cytokines (IL-6, IL-17, TNF-α) and upregulation of anti-inflammatory IL-10, thereby mitigating chronic inflammatory conditions, including colitis (Jin et al. 2025). Antioxidant capacity is further demonstrated through the synthesis of multiple antioxidant molecules by *B. longum*, effectively scavenging reactive oxygen species and reducing oxidative stress-induced intestinal epithelial damage (Gagnon et al. 2015).

Compared with giant pandas at the onset of old age (20–25 years), the elevated abundance of *L. intestinalis* in long-lived elderly individuals suggests potential contributions to immune and metabolic homeostasis. Elevated *L. intestinalis* abundance correlates with rejuvenation of T-cell subset composition and negatively correlates with host blood glucose levels. These correlations indicate possible enhancement of glucose metabolism through improved insulin sensitivity and glucose utilization, thereby preventing age-related metabolic disorders (Wang et al. 2023). Although molecular pathways underlying these effects require further investigation, *lactobacilli* are widely recognized for intestinal health benefits through lactate production. Lactate production lowers intestinal pH and inhibits pathogens, establishing this genus as a critical probiotic link between gut microbiota and host immunometabolic homeostasis (Huang et al. 2024).

*R. gnavus* exhibits complex ecological roles. Carbohydrate fermentation capacity of this Ruminococcaceae member contributes to energy and nutrient provision (Crost et al. 2023; Nooij et al. 2025). Excessive proliferation, however, is frequently associated with suboptimal health states, including metabolic, inflammatory, and neurological disorders (Henke et al. 2019; He et al. 2025). Recent metagenomic studies reveal a significant correlation between *R. gnavus* and advanced age (≥90 years) in multiple Chinese and European populations, potentially promoting healthy aging through primary bile acid metabolism modulation (Andreu-Sánchez et al. 2025). Elevated *R. gnavus* abundance in long-lived giant pandas occurred without inducing significant inflammation, possibly attributable to rich and balanced microbial diversity within their intestinal environment. Collective insights from human centenarians and long-lived giant pandas underscore the importance of beneficial microbiota enrichment and ecological balance maintenance as potential intervention strategies for promoting longevity.

### Comparative microbiome perspectives on aging and longevity across species

To understand how beneficial bacteria may contribute to giant panda longevity, we systematically compared microbiome features across phylogenetically diverse species in which microbial communities have been linked to lifespan regulation (Supplementary Table S8). This cross-species analysis encompasses both naturally long-lived species and short-lived model organisms where experimental interventions extend relative lifespan, revealing conserved mechanisms alongside species-specific adaptations in microbiome-mediated longevity.

*Lactobacillus* and *Bifidobacterium* enrichment appears as a phylogenetically conserved longevity signature across humans, naked mole-rats, honey bee queens, and probiotic-supplemented *Caenorhabditis elegans* (Debebe et al. 2017; Donato et al. 2017; Anderson et al. 2018; Sato et al. 2021). Mechanistically, microbial modulation of insulin/IGF-1 signaling represents an evolutionarily conserved pathway, with *Bacillus subtilis* extending *C. elegans* lifespan through insulin-like receptor downregulation and queen bee microbiota prolonging worker lifespan via similar mechanisms (Donato et al. 2017; Wang et al. 2024), suggesting ancient microbial strategies for aging regulation.

Substantial variation exists across species, complicating the establishment of universal principles. Alpha diversity patterns diverge depending on host ecology: diversity increases in human centenarians and killifish receiving young microbiota (Smith et al. 2017; Pang et al. 2023) but decreases in dietary specialists, including pandas and bee queens (Anderson et al. 2018; Zhang et al. 2018). This suggests optimal microbiome structure may depend on host-specific constraints, with functional composition potentially more predictive of longevity than taxonomic diversity metrics. Experimental manipulations demonstrate causal potential: young-donor fecal transplants extended killifish lifespan by 41% (Smith et al. 2017), though whether naturally occurring microbiome shifts similarly drive aging phenotypes requires further investigation.

Long-lived pandas show notable compositional parallels to other longevity systems despite ecological divergence. Enrichment of *A. muciniphila*, *B. longum*, and *L. intestinalis* mirrors patterns in centenarians, with *A. muciniphila* potentially contributing to reduced inflammation through enhanced barrier integrity (Reunanen et al. 2015; Sato et al. 2021). Elevated *R. gnavus*, which associates with inflammation in younger populations but increases with age in centenarians (Pang et al. 2023), suggests context-dependent functional roles possibly mediated by age-related metabolic shifts. These compositional similarities across phylogenetically distant long-lived species indicate dietary specialization shapes microbiome assembly without precluding convergence on longevity-associated taxa. Distinguishing conserved microbial functions from host-specific adaptations will require integrative approaches combining metagenomics, metabolomics, and metatranscriptomics across comparative systems.

### Age-associated CAZyme enrichment in giant panda gut microbiomes

Cross-sectional metagenomic profiling revealed age-associated CAZyme remodeling in giant panda gut microbiomes. Long-lived pandas exhibited cellulolytic enzyme enrichment (β-glucosidase GH3, endoglucanase GH5, exoglucanase GH9), reflecting adaptive responses to lifelong bamboo consumption requiring continuous polysaccharide degradation. Given bamboo's low nutritional density and high fiber content, efficient cellulose fermentation remains essential for energy acquisition throughout the panda's lifespan. In contrast, *Sardinian centenarians* exhibited enhanced glycolytic and SCFA fermentation capacity despite reduced fiber-degrading enzymes (Wu et al. 2019), reflecting dietary context: humans consume diverse omnivorous diets, whereas pandas depend on sustained fiber-processing throughout life.

Fiber fermentation mediates critical host–microbiome metabolic interactions during aging. Cellulolytic bacteria degrade complex polysaccharides into fermentable oligosaccharides and acetate. Secondary fermenters utilize these substrates to produce propionate and butyrate, typically yielding acetate:propionate:butyrate ratios of ∼60:25:15. However, conversion efficiency varies considerably based on microbiome composition. Butyrate serves as a major colonocyte energy source and inhibits histone deacetylases, inducing antioxidant and cytoprotective gene expression (Dalile et al. 2019). These microbial metabolites function as central regulators in host metabolic homeostasis, modulating glucose and lipid metabolism, inflammatory responses, and oxidative stress pathways (Agus et al. 2021). In acarbose-treated mice, fecal SCFA concentrations predicted extended lifespan regardless of sex and site (Smith et al. 2019). Geographically distinct centenarian populations demonstrated robust SCFA-producing capacity through glycolytic and fermentation pathways (Wu et al. 2019; Pang et al. 2023), characteristics associated with exceptional human longevity.

Comparative metagenomic analyses showed that cellulosome-harboring *Ruminococcus* species, abundant in ancient humans and contemporary hunter-gatherers, are depleted in industrialized populations (Moraïs et al. 2024), indicating reduced fiber-degrading capacity in modern gut microbiomes. The sustained cellulolytic capacity in aged pandas contrasts markedly with this depletion in modern humans, suggesting plant polysaccharide fermentation may play important roles in maintaining metabolic function during mammalian aging.

### Age-associated BGC enrichment patterns

BGCs encode enzymatic machinery for biosynthesis of structurally diverse specialized metabolites mediating microbe–host interactions (Donia and Fischbach 2015; Wang et al. 2019). Giant pandas exhibited age-dependent BGC diversity expansion—an approximately 5-fold increase from adolescents to advanced adults (*P* < 0.001)—paralleling human centenarians, where enhanced biosynthetic repertoires associate with exceptional longevity (Biagi et al. 2016; Liu et al. 2024). This expansion primarily involved pathways addressing 3 hallmarks of aging: immune dysfunction, oxidative stress, and metabolic dysregulation.

Multiple antimicrobial and immunomodulatory BGC families showed age-associated accumulation. NRPS and RiPP systems, producing peptide-based specialized metabolites including antimicrobial peptides (Walsh 2016; Montalbán-López et al. 2021), may compensate for declining adaptive immunity by enhancing bacteriocin production and maintaining colonization resistance (Cullen et al. 2015; Donia and Fischbach 2015). Similarly, NI-siderophore BGCs were enriched in aged pandas, with these iron-chelating compounds limiting pathogen iron availability (Zhu et al. 2020)—a mechanism increasingly relevant when tissue iron dysregulation occurs during aging. Antioxidant defense systems also expanded with age. Arylpolyene BGCs showed 1.8-fold enrichment (*P* < 0.01), with these membrane-associated pigments protecting against reactive oxygen species (Schöner et al. 2016). Terpene biosynthetic genes increased progressively, including *A. muciniphila*-encoded squalene/phytoene synthases producing anti-inflammatory triterpenoids (Liu et al. 2024). Centenarian gut microbiomes similarly exhibit enhanced oxidoreductase capacity and terpene-producing bacteria (Ge et al. 2021; Wu et al. 2022; Liu et al. 2024).

Indole biosynthetic genes exhibited negative age correlation, suggesting tryptophan catabolism remodeling (Roager and Licht 2018). Despite genetic depletion, indole-3-propionic acid production can remain elevated in long-lived individuals through enhanced metabolic efficiency, associating with increased survival via aryl hydrocarbon receptor activation, oxidative stress reduction, and intestinal barrier enhancement (Wilmanski et al. 2021). Indole derivatives also alleviate neurodegeneration through GPR30/AMPK/SIRT1 pathway activation (Yin et al. 2023). This age-dependent BGC expansion reflects biosynthetic genetic potential consistent with microbial drift toward compositional uniqueness, a longevity-associated hallmark (Biagi et al. 2016; Wilmanski et al. 2021), with enriched pathways potentially enabling gut microbiome functional stability during late-life aging (Franceschi et al. 2018; Ghosh et al. 2022). Supporting this notion, fecal microbiota transplantation from long-lived donors improved metabolic parameters and extended lifespan in preclinical models (Bárcena et al. 2019).

Critically, although BGC analysis reveals genetic potential for specialized metabolite production, actual synthesis rates, bioavailability, and biological activities require validation through targeted metabolomics quantifying microbially derived metabolites in fecal and plasma samples. Functional assays are needed to confirm predicted bioactivities and establish causal contributions to longevity-associated phenotypes.

### Translational potential of giant panda-derived probiotics

Given the enrichment of LAB observed in long-lived giant pandas, strains isolated from giant panda feces (such as *L. johnsonii*, *L. reuteri*, *P. acidilactici*, *L. intestinalis*) demonstrate promising food application potential (Peruzzolo et al. 2025). *L. johnsonii* and *L. reuteri* appear on the National Health Commission of China's List of Bacterial Species Approved for Food Use, with all strains exhibiting non-hemolytic properties and antibiotic sensitivity profiles meeting preliminary safety criteria.

Multiple biological functions have been demonstrated in animal and cell models. *L. johnsonii* strains antagonize pathogen colonization, modulate mucosal and systemic immunity, alleviate chronic inflammation, ameliorate metabolic disorders, and enhance epithelial barrier function, contributing to host homeostasis maintenance (Fonseca et al. 2017; Zou et al. 2020; Yuan et al. 2023; Zhang et al. 2023; Arzola-Martínez et al. 2024). *P. acidilactici*, the most frequently isolated species, exhibits significant immunomodulatory and metabolic effects. Growth promotion and immune enhancement occur in aquatic animal models (Yavorov-Dayliev et al. 2023; Zhao et al. 2024; Woo et al. 2025). *P. acidilactici* MA18/5M improves intestinal barrier function while modulating tight junction and immune-related gene expression (Soto-Dávila et al. 2024); *P. acidilactici* B49 enhances fish intestinal lysozyme activity and mucosal immunoglobulin levels, reduces TGF-β expression, and augments peripheral blood lymphocyte phagocytosis (Wang et al. 2025). In mammalian models, canine-derived *P. acidilactici* (GLP06) significantly increases antioxidant enzyme activity, reduces lipid peroxidation products, elevates intestinal IgA and IgG, increases beneficial microbiota abundance, and enhances fecal SCFA production (Zhao et al. 2024).

*L. intestinalis*, enriched in long-lived giant pandas despite traditionally low abundance in human intestines, exhibits unique immunomodulatory mechanisms. In DSS-induced colitis models, epithelial cell signaling modulation reduces pro-inflammatory SAA1/2 production, indirectly suppressing Th17 differentiation (Wang et al. 2023). Synergistic metabolism with host aldehyde dehydrogenase enhances intestinal retinoic acid generation, activating RA receptor-mediated anti-inflammatory effects. Clinical studies have demonstrated a negative correlation between *L. intestinalis* abundance and inflammatory pathways in ulcerative colitis patients, specifically showing an inverse relationship with the C/EBPA-SAA1/2-Th17 axis (Wang et al. 2023), thus supporting the clinical relevance of our findings.

Although these mechanistic insights and established strain repositories provide promising foundations for therapeutic development, translating animal-derived probiotics to human applications requires systematic evaluation of strain stability, cultivability under industrial conditions, and host-specific colonization patterns (Hill et al. 2014; Zmora et al. 2018). Follow-up studies are currently underway to assess each isolate's stability and cultivability under conditions relevant to human probiotic use. Key experiments involve testing the strains' viability in simulated gastrointestinal environments—for instance, exposure to gastric acid and bile salts—because an effective probiotic must survive the harsh conditions of the human gut (Makinen et al. 2012). We are also examining the survival of these LAB during processing and storage, as a sufficient number of viable bacteria must be delivered at consumption for efficacy (Hill et al. 2014). Equally important, we are verifying that these strains can be readily cultured and scaled up in standard fermentation media without loss of function. Robust growth, genetic stability, and safety in an industrial setting are crucial criteria for any candidate probiotic (Anandharaj et al. 2017). By systematically addressing stability, viability, and manufacturability, we aim to determine the practical probiotic potential of the panda-derived strains. This approach will clarify whether their unique origins—from an endangered species with exceptional longevity—can be translated into viable probiotic applications in human or veterinary medicine, thereby laying the groundwork for future preclinical or clinical development.

### Methodological limitations

Our culture-dependent isolation approach using MRS agar supplemented with calcium carbonate has important methodological limitations. The calcium dissolution zone-based selection criterion systematically favors acidogenic bacterial taxa, potentially excluding species that do not form prominent dissolution zones—including certain *Bifidobacterium* spp. and subpopulations of *Lacticasei bacillus*—that may possess unrecognized probiotic functions (Derrien et al. 2022). Furthermore, we observed an inverse relationship between metagenomic abundance of *L. intestinalis* (peak abundance in adolescent giant pandas) and our isolation success (only 21/98 isolates from long-lived individuals), suggesting age-dependent shifts in bacterial culturability. This culture-dependent bias is well-documented in gut microbiome research (Lagier et al. 2015; Forster et al. 2019), with studies demonstrating that cultivation approaches using single modifications to culture media recover only 42% of species detected in original stool samples (Armetta et al. 2025). Future studies should employ complementary multiple-media culturomics strategies, including selective media for non-acidogenic LAB and age-matched sampling across all life stages. Such approaches would enable comprehensive characterization of functional diversity in the giant panda gut microbiome and validation of age-specific distribution patterns observed in metagenomic analyses.

In summary, stage-specific succession patterns of the gut microbiome across giant panda life stages were revealed, with long-lived individuals harboring distinct enterotype structures. Co-enrichment of putative longevity-associated probiotics (*A. muciniphila*, *B. longum*, *L. intestinalis*) and metabolic pathways (fatty acid synthesis and glyoxylate cycle) characterized the gut microbiomes of long-lived individuals. Age-dependent evolution of microbial functional profiles corresponded with physiological changes during host aging, demonstrating co-adaptive dynamics between host and microbiome throughout senescence. Multiple putative longevity-associated probiotic strains (*L. johnsonii*, *L. reuteri*, *P. acidilactici*, and *L. intestinalis*) were successfully isolated from giant pandas and subjected to comprehensive safety assessments. This study provides important theoretical foundations for elucidating host–microbe synergistic mechanisms in aging, while establishing scientific groundwork for developing functional probiotics promoting healthy longevity. Future investigations will validate candidate strain efficacy through mechanistic analyses and animal models, advancing translational applications in microecological therapeutics and age-related disease prevention.

## Supplementary Material

zoaf074_Supplementary_Data

## Data Availability

Host-removed raw metagenomic sequencing data have been deposited in the NCBI Sequence Read Archive (SRA) under BioProject accession number PRJNA1338869. All other data supporting the findings of this study are available from the corresponding author upon reasonable request.
