# Supplementary material for: Gut microbiome-driven longevity mechanisms in giant pandas: age-associated gut microbial shifts, functional pathways, and probiotic insights
Source: Curr Zool. 2025 Nov 21;72(5):616–38. doi: 10.1093/cz/zoaf074 (PMC13690582; doi:10.1093/cz/zoaf074)
Supplement: Supplementary file 1 [file zoaf074_supplementary_data.zip › Figure S1.pdf]

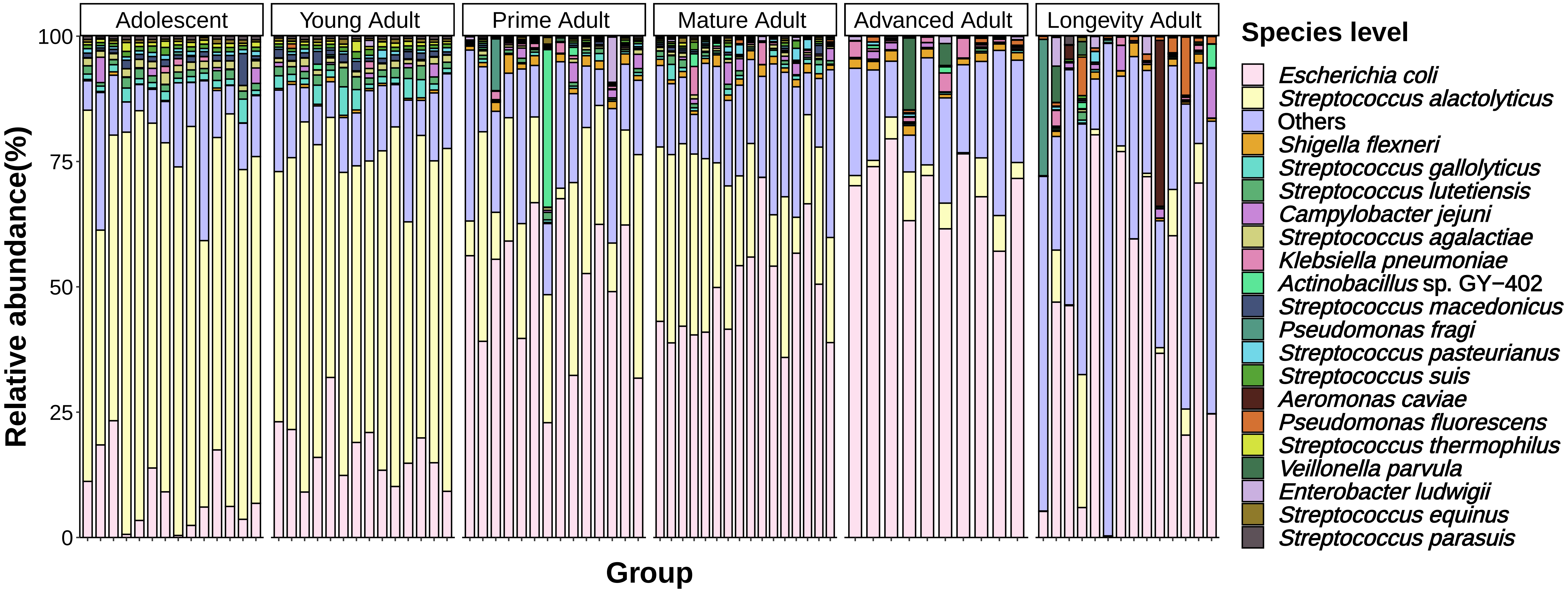
Adolescent
Young Adult
Prime Adult
Mature Adult
Advanced Adult
Longevity Adult
Relative abundance(%)
100
75
50
25
0
Group
Species level
Escherichia coli
Streptococcus alactolyticus
Others
Shigella flexneri
Streptococcus gallolyticus
Streptococcus lutetiensis
Campylobacter jejuni
Streptococcus agalactiae
Klebsiella pneumoniae
Actinobacillus sp. GY−402
Streptococcus macedonicus
Pseudomonas fragi
Streptococcus pasteurianus
Streptococcus suis
Aeromonas caviae
Pseudomonas fluorescens
Streptococcus thermophilus
Veillonella parvula
Enterobacter ludwigii
Streptococcus equinus
Streptococcus parasuis
