# Supplementary material for: Gut microbiome-driven longevity mechanisms in giant pandas: age-associated gut microbial shifts, functional pathways, and probiotic insights
Source: Curr Zool. 2025 Nov 21;72(5):616–38. doi: 10.1093/cz/zoaf074 (PMC13690582; doi:10.1093/cz/zoaf074)
Supplement: Supplementary file 1 [file zoaf074_supplementary_data.zip › Figure S2.pdf]

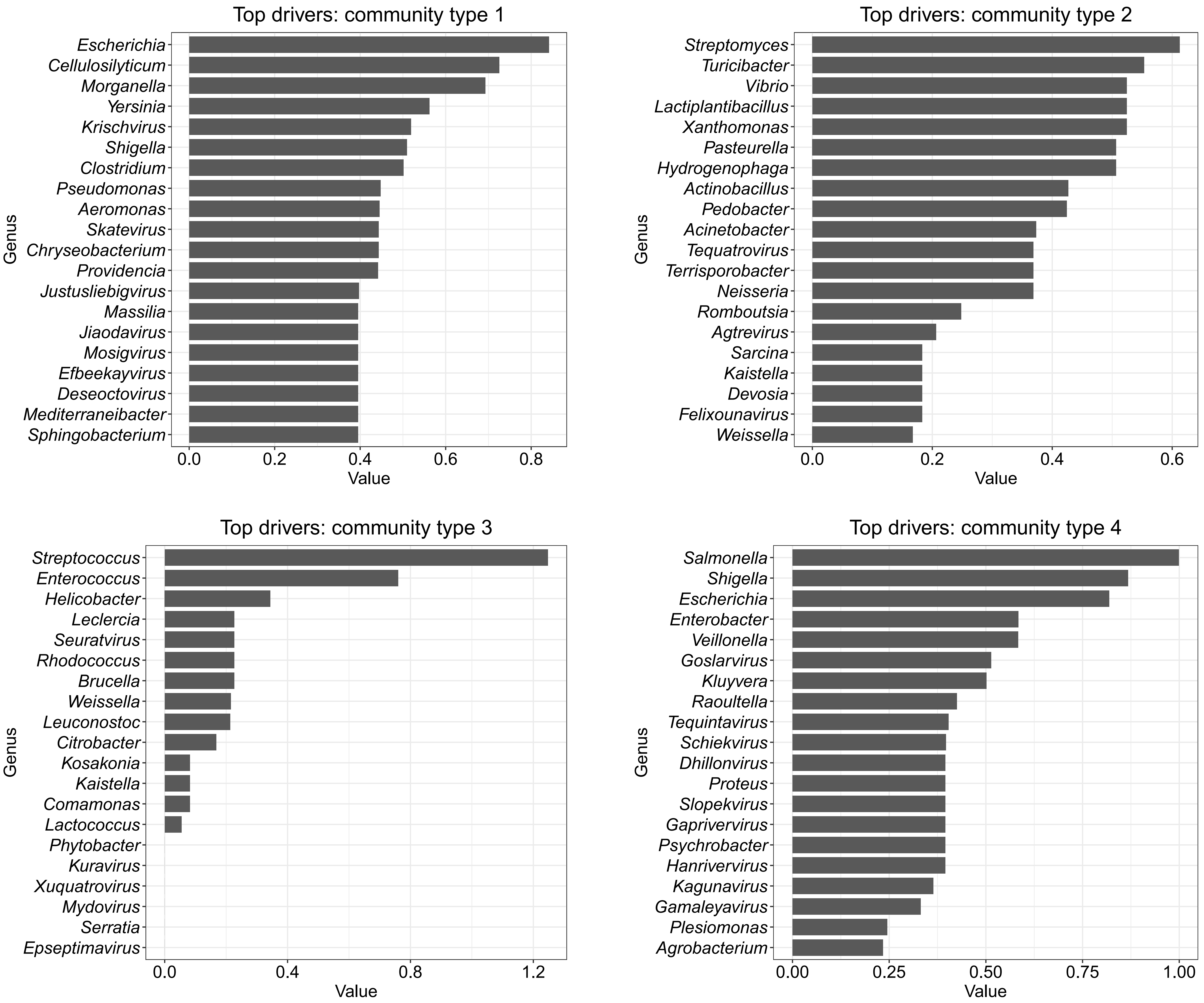
Top drivers: community type 1
Escherichia
Cellulosilyticum
Morganella
Yersinia
Krischvirus
Shigella
Clostridium
Pseudomonas
Aeromonas
Skatevirus
Chryseobacterium
Providencia
Justusliebigvirus
Massilia
Jiaodavirus
Mosigvirus
Efbeekayvirus
Deseoctovirus
Mediterraneibacter
Sphingobacterium
Genus
Value
0.0
0.2
0.4
0.6
0.8
Top drivers: community type 2
Streptomyces
Turicibacter
Vibrio
Lactiplantibacillus
Xanthomonas
Pasteurella
Hydrogenophaga
Actinobacillus
Pedobacter
Acinetobacter
Tequatrovirus
Terrisporobacter
Neisseria
Romboutsia
Agtrevirus
Sarcina
Kaistella
Devosia
Felixounavirus
Weissella
Genus
Value
0.0
0.2
0.4
0.6
Top drivers: community type 3
Streptococcus
Enterococcus
Helicobacter
Leclercia
Seuratvirus
Rhodococcus
Brucella
Weissella
Leuconostoc
Citrobacter
Kosakonia
Kaistella
Comamonas
Lactococcus
Phytobacter
Kuravirus
Xuquatrovirus
Mydovirus
Serratia
Epseptimavirus
Genus
Value
0.0
0.4
0.8
1.2
Top drivers: community type 4
Salmonella
Shigella
Escherichia
Enterobacter
Veillonella
Goslarvirus
Kluyvera
Raoultella
Tequintavirus
Schiekvirus
Dhillonvirus
Proteus
Slopekvirus
Gaprivervirus
Psychrobacter
Hanrivervirus
Kagunavirus
Gamaleyavirus
Plesiomonas
Agrobacterium
Genus
Value
0.00
0.25
0.50
0.75
1.00
